# SQSTM1 promotes selective autophagic degradation of multiple PRRSV proteins by recruiting E3 ligase TRAF6 to inhibit viral replication

**DOI:** 10.1186/s13567-026-01887-8

**Published:** 2026-11-14

**Authors:** Hong Duan, Fengxia Zhang, Yunshuo Lu, Ziheng Zhang, Min Wang, Chaoyu Yang, Jiahui Ren, Jianli Li, Dongliang Li, Linyang Yu, Lu Chen, Jun Zhao, Yongkun Du, Gaiping Zhang, Angke Zhang

**Affiliations:** 1 College of Veterinary Medicine, Henan Agricultural University, Zhengzhou, 450046 China; 2 International Joint Research Center of National Animal Immunology, College of Veterinary Medicine, Henan Agricultural University, Zhengzhou, 450046 China; 3 Ministry of Education Key Laboratory for Animal Pathogens and Biosafety, College of Veterinary Medicine, Henan Agricultural University, Zhengzhou, 450046 China; 4 Longhu Laboratory of Advanced Immunology, Zhengzhou, 450046 China

**Keywords:** PRRSV, SQSTM1, autophagic degradation, antiviral activity

## Abstract

Porcine reproductive and respiratory syndrome (PRRS) is an economically important disease that endangers the global pig industry and is caused by porcine reproductive and respiratory syndrome virus (PRRSV). Although previous studies have reported that PRRSV hijacks autophagy to facilitate its replication, the role of selective autophagy in PRRSV propagation remains largely unclear. Here, we identified the autophagic cargo receptor SQSTM1 as a novel host restriction factor against PRRSV. SQSTM1 overexpression significantly suppresses PRRSV replication, while SQSTM1 knockdown promotes viral proliferation. Moreover, SQSTM1 overexpression enhances the autophagic degradation of multiple PRRSV proteins, including Nsp5, Nsp12, GP4, and GP5. We further selected GP4 and GP5 as target proteins to explore the molecular mechanism by which SQSTM1 degrades PRRSV proteins. SQSTM1 promotes the trafficking of GP4 to LAMP2-positive lysosomes for autophagy‑lysosome-dependent degradation. SQSTM1 directly interacts with both GP4 and GP5, and recruits the host E3 ubiquitin ligase TRAF6 to enhance the polyubiquitination of these two viral proteins. Both the LC3-interacting region (LIR) and ubiquitin-associated (UBA) domains of SQSTM1 are required for SQSTM1-mediated inhibition of PRRSV replication. Further mechanistic validation showed that TRAF6 mediates K63-linked polyubiquitination of GP4 at the K115 residue and GP5 at the K59 residue. These ubiquitination modifications are indispensable for the binding of GP4/GP5 to SQSTM1 and their subsequent autophagic degradation. TRAF6 knockdown abrogates SQSTM1-induced degradation of PRRSV Nsp5, Nsp12, GP4, and GP5, and eliminates the antiviral activity of SQSTM1, indicating that SQSTM1 and TRAF6 synergistically degrade viral proteins and constrain PRRSV replication. The present study demonstrates the critical role of selective autophagy in host defense against PRRSV infection. These findings provide novel insights for the development of targeted anti-PRRSV drugs and vaccines.

## Introduction

Porcine reproductive and respiratory syndrome (PRRS), caused by porcine reproductive and respiratory syndrome virus (PRRSV), remains one of the major concerns in the global swine industry [1]. PRRSV infection results in reproductive failure in pregnant sows and severe respiratory disease with high fever in pigs across all age groups [2]. PRRSV is an enveloped, positive-sense, single-stranded RNA virus belonging to the family *Arteriviridae* in the order *Nidovirales* [3].

The PRRSV genome is approximately 15 kb in length and contains at least 10 open reading frames (ORFs). ORFs 1 a and 1 b encode two large polyproteins, which are subsequently cleaved into fourteen nonstructural proteins (Nsp1α, Nsp1β, Nsp2–12) responsible for genome replication and transcription [4, 5]. ORFs 2–7, 2 b and 5 a encode eight viral structural proteins, including three N-glycosylated minor envelope proteins (GP2a, GP3 and GP4), the major envelope glycoprotein GP5 (which forms a heterodimer with the non-glycosylated membrane protein M) [6], the highly immunogenic nucleocapsid protein (N), and the non-glycosylated E and GP5a proteins. The three main structural proteins (M, N and GP5) are required for virion formation and viral infectivity; the absence of any of these proteins results in failure to produce infectious virions. In contrast, the minor envelope proteins (GP2a, GP3 and GP4) are essential only for viral infectivity [7, 8]. The E protein has a single cross-helical structure and ion channel activity, and is a minor structural protein of viral particles [9, 10]. Studies have shown that minor envelope proteins (GP2a to GP4) facilitate PRRSV spread and viral yield in host cells [11, 12]. Notably, GP4 can elicit protective neutralizing antibodies in pigs [13–16]. GP4 colocalizes with the critical PRRSV receptor cluster of differentiation 163 (CD163) and the host protein heat shock protein family A member 8 (HSPA8), to mediate viral attachment and entry into host cells [17, 18]. GP5-M heterodimers can bind to sialoadhesin (Sn), which is sufficient to mediate viral binding and internalization [19, 20]. Palmitoylation of the envelope proteins GP5 and M is essential for PRRSV replication [21]. Specifically, the GP2a, GP3, GP4, and GP5 proteins form a heterotetrameric complex required for their transport from the endoplasmic reticulum (ER) to the Golgi apparatus in infected cells prior to virion assembly [22]. Despite its reported role as a major determinant of viral cellular tropism, little research has explored GP4’s interaction with host proteins during PRRSV replication.

Cellular autophagy is widely acknowledged as a conserved cellular process that clears damaged proteins and organelles, as well as eliminates invading pathogens [23]. Autophagy can be categorized into nonselective or selective types. Nonselective autophagy is implicated in the response to nutritional deprivation, during which cytoplasmic components are nonspecifically sequestered into autophagosomes. In contrast, selective autophagy promotes the degradation of aggregated proteins or damaged organelles through cargo receptor-mediated recognition [24, 25]. Prominent autophagy receptors include sequestosome 1 (SQSTM1)/p62, neighbor of BRCA1 (NBR1), optineurin (OPTN), calcium-binding and coiled-coil domain-containing protein 52 (CALCOCO2/NDP52), and Toll-interacting protein (TOLLIP) [26–29]. These receptors bind to their targets via ubiquitin or galectin (LGALS) tags and to phagophore-anchored microtubule-associated protein 1 light chain 3 (LC3) molecules through LC3-interacting regions (LIRs), which is followed by lysosome-mediated degradation. Among these receptors, SQSTM1 is a classical selective autophagy receptor characterized by a modular structure encompassing a Phox and Bem1p (PB1) domain, a ZZ-type zinc finger motif (ZZ), a ubiquitin-associated (UBA) domain, and an LC3-interacting region (LIR) [30].

Autophagy is a crucial cellular defense mechanism against invading viruses. Host antiviral effects are often associated with the selective recognition of incoming virus particles by autophagy receptors, followed by the engulfment and degradation of these particles. Polyubiquitination of substrate proteins has been established as a marker for selective autophagic recognition and degradation, indicating that a ubiquitin-dependent sensor system determines substrate specificity [31]. For viruses infecting domestic animals, previous studies have demonstrated that SQSTM1 contributes to the inhibition of porcine deltacoronavirus [32], porcine epidemic diarrhea virus [33], infectious bursal disease virus [34], avian metapneumovirus subgroup C [35], and avibirnavirus [36]. Previous studies have demonstrated that PRRSV hijacks host autophagic flux to facilitate its own replication [37–39]. In addition, PRRSV Nsp2, Nsp3, and Nsp5 proteins are involved in the induction of cellular autophagy [40–43]. On the other hand, some host proteins can exert anti-PRRSV activity by activating autophagy to degrade key viral components. For instance, DEAD-box RNA helicase 27 (DDX27) suppresses PRRSV replication by catalyzing the autophagic degradation of viral GP2a and inducing interferon-β production [44]. Proteasome 20 S subunit beta 1 (PSMB1) recruits the autophagy receptor NBR1 and the E3 ubiquitin ligase STUB1 to degrade Nsp12 via the autophagy-lysosomal pathway [45]. PSMB4 promotes the autophagic degradation of PRRSV Nsp1α to facilitate the production of interferon and thus inhibits viral replication [46]. Despite this, the association between selective autophagy and PRRSV infection remains poorly understood, and whether autophagic cargo receptor proteins participate in the regulation of PRRSV replication remains unknown.

The present study investigated the role of selective autophagy during PRRSV replication. Results showed that overexpression of the selective autophagy receptor SQSTM1 inhibited PRRSV replication, whereas SQSTM1 knockdown promoted viral replication. SQSTM1 was found to trigger autophagic degradation of PRRSV proteins Nsp5, Nsp12, GP4 and GP5. Further mechanistic studies focusing on GP4 and GP5 demonstrated that SQSTM1 interacts with both PRRSV GP4 and GP5 and recruits the host E3 ubiquitin ligase TNF receptor-associated factor 6 (TRAF6) to drive degradation of these viral proteins via the autophagy–lysosomal pathway. TRAF6-catalyzed K63-linked polyubiquitination of GP4 and GP5 is required for their interaction with SQSTM1 and subsequent autophagic degradation. TRAF6 knockdown abrogated SQSTM1-mediated degradation of Nsp5, Nsp12, GP4 and GP5, as well as the anti-PRRSV activity of SQSTM1. Collectively, these findings reveal that selective autophagy plays a critical role in host defense against PRRSV infection.

## Materials and methods

### Cells and viruses

Marc-145 cells—the most commonly used cell line permissive to PRRSV replication in vitro—and human embryonic kidney 293T (HEK-293T) cells were cultured in Dulbecco's modified Eagle's medium (DMEM, KeyGEN, KGL1211-500) supplemented with 10% fetal bovine serum (FBS, Gibco, 10099141C), 100 μg/mL streptomycin and 100 IU/mL penicillin (Solarbio, P7630), at 37 °C in a 5% CO_2_ humidified atmosphere. Porcine alveolar macrophages (PAMs), the natural host cells for PRRSV in vivo, were preserved in our lab and were cultured in RPMI-1640 medium containing 10% FBS, 100 μg/mL streptomycin, and 100 IU/mL penicillin, at 37 °C in a 5% CO_2_ humidified atmosphere.

The following PRRSV strains were used in the present study: GD-HD (GenBank accession number KP793736.1), SD16 (GenBank accession number JX087437.1), the PRRSV-2 prototype strain VR-2332 (GenBank accession number EF536003.1), the JXA1 strain (GenBank accession number EF112445.1), and the NADC30-like strain (GenBank accession number MH651738.1). Of note, GD-HD was used in most experiments unless otherwise specified and is simply referred to as "PRRSV" herein. All PRRSV strains were propagated and titrated in Marc-145 cells, then stored at −80 °C as viral stocks for subsequent use.

### Antibodies and biochemical reagents

Mouse anti-GP4, anti-N protein monoclonal antibodies (mAbs), and anti-GP5 polyclonal antibody (pAb) were prepared in our laboratory. Rabbit anti-SQSTM1 polyclonal antibody (pAb, 18420-1-AP), rabbit anti-GFP pAb (50430-2-AP), and rabbit anti-TRAF6 pAb (83535-2-RR) were obtained from Proteintech Group. Mouse anti-HA tag monoclonal antibody (mAb, AF2858) and rabbit anti-Myc tag mAb (AG0305), mouse anti-α-tubulin mAb (AF2827), horseradish peroxidase (HRP)-conjugated goat anti-mouse IgG antibody (A0192), and HRP-conjugated goat anti-rabbit IgG antibody (A0208) were all obtained from Beyotime Biotechnology. Alexa Fluor 488-conjugated goat anti-rabbit IgG (H&L, ab150077) and Alexa Fluor 594-conjugated goat anti-mouse IgG (H&L, ab150116) were both obtained from Abcam.

The autophagy-lysosomal inhibitor chloroquine (CQ, HY-17589 A), and the autophagy activator rapamycin (Rap, HY-10219) were both purchased from MedChemExpress.

### Plasmid construction and transfection

PRRSV non-structural or structural genes Nsp3, GP2a, GP3, GP5 and M were inserted into the pEGFP-C1 vector to construct GFP-tagged recombinant plasmids pEGFP-Nsp3, pEGFP-GP2a, pEGFP-GP3, pEGFP-GP5, and pEGFP-M. The Nsp1α, Nsp1β, Nsp2, Nsp4, Nsp5, Nsp9, Nsp11, Nsp12, GP4, GP5, N and E genes were inserted into the pCAGGS-HA vector to construct HA-tagged recombinant plasmids. The porcine TRAF6 gene was cloned into the pCMV-3 × Flag eukaryotic expression plasmid to generate the pCMV-TRAF6-3 × Flag recombinant plasmid. The porcine SQSTM1 gene was cloned into the pCMV-Myc vector to construct the recombinant plasmid pCMV-SQSTM1-Myc. Additionally, His-Ubiquitin (Ub)-K63, HA-Ub-K48, HA-Ub-K63, and HA-Ub-K63R plasmids were preserved in our laboratory. The GFP-LC3 reporter plasmid was purchased from Addgene (#11546). The mutant plasmids pCAGGS-GP4-K13R-HA, pCAGGS-GP4-K24R-HA, pCAGGS-GP4-K35R-HA, pCAGGS-GP4-K65R-HA, pCAGGS-GP4-K112R-HA, pCAGGS-GP4-K115R-HA, pCAGGS-GP4-K139R-HA, as well as pCAGGS-GP5-K4R-HA, pCAGGS-GP5-K59R-HA, pCAGGS-GP5-K129R-HA, and pCAGGS-GP5-K149/163/166/177R-HA were all constructed in our laboratory. Autophagy receptor-related plasmids, including pCMV-SQSTM1-3 × Myc, pCMV-NDP52-3 × Myc, pCMV-OPTN-3 × Myc, pCMV-NBR1-3 × Myc, pCMV-TOLLIP-3 × Myc, and pCMV-BNIP3L-3 × Myc, were constructed in our laboratory. SQSTM1 mutant plasmids (pCMV-SQSTM1-ΔUBA-Myc, pCMV-SQSTM1-ΔPB1-Myc, pCMV-SQSTM1-ΔLIR-Myc, and pCMV-SQSTM1-ΔZZ-Myc) were constructed in our laboratory. Recombinant plasmids were transfected into Marc-145 cells using X-tremeGENE™ HP DNA Transfection Reagent (Roche Diagnostics, 06366236001) and into HEK-293T cells using polyethyleneimine (PEI) Transfection Reagent (Proteintech Group, PR40001) following the manufacturers’ instructions.

### Co-immunoprecipitation (Co-IP) and western blot analysis

HEK-293 T cells were transfected with HA-GP4 (1 μg) or HA-GP5 (1 μg) plasmids together with Myc-SQSTM1 (1 μg) or Flag-TRAF6 (1 μg) plasmids. At 48 hours post-transfection (hpt), cells were harvested and lysed in NP40 lysis buffer supplemented with 1% phenylmethylsulfonyl fluoride (PMSF, Beyotime, ST505) for 30 min on ice. After centrifugation at 13000 × *g* for 10 min at 4 °C, the supernatants were collected and co-incubated with Protein G MagBeads (GenScript, L00277) pre-coated with anti-HA (1.5 μg per sample), anti-Myc (1.5 μg per sample), or anti-Flag (1.5 μg per sample) tag mAbs at room temperature (RT) for 2 h on a rotor. The magnetic beads were then precipitated using a strong magnet, and the supernatants were discarded. The beads were washed three times with 1 mL of phosphate-buffered saline (PBS), after which 2 × SDS loading buffer was added. Samples were boiled for 5 min and subsequently subjected to western blot analysis.

Proteins were separated by SDS–polyacrylamide gel electrophoresis (SDS-PAGE) and then transferred to polyvinylidene fluoride (PVDF) membranes (Millipore, ISEQ00010). After blocking with 2.5% non-fat dry milk (Solarbio, D8340) in PBST at 4 °C overnight, the membranes were probed with anti-HA (1:1000), anti-Myc (1:1000), or anti-Flag (1:1000) mAbs as primary antibodies at RT for 1 h, followed by three washes with PBST. The membranes were then incubated with HRP-conjugated goat anti-mouse IgG (H&L) (1:5000) or anti-rabbit IgG (H&L) (1:5000) as secondary antibodies for 1 h at RT, and washed again three times with PBST. Target bands were visualized using an ECL chemiluminescent reagent (Tanon, 180–5001).

### TCID_50_ assay

After the indicated treatments, cell culture supernatants were collected, and viral titers were detected as previously described [47]. Briefly, supernatant samples were serially diluted 10-fold and then added to monolayer Marc-145 cells with 80% confluence (100 μL/well), followed by incubation at 37 °C for approximately 1 h. The cells were washed with PBS (100 μL/well) and subsequently cultured in 3% FBS + DMEM (175 μL/well). Cytopathic effects (CPE) were monitored continuously for 5–7 days, and viral titers of the different supernatant samples were calculated as the 50% tissue culture infective dose (TCID_50_) using the Reed‒Muench method.

### Laser confocal detection

To determine whether HA-GP4 or HA-GP5 (0.25 μg) colocalizes with Myc-SQSTM1 or Flag-TRAF6, HEK-293 T cells were co-transfected with pCAGGS-GP4-HA (0.25 μg), pCAGGS-GP5-HA (0.25 μg), and either pCMV-SQSTM1-Myc (0.25 μg) or pCMV-TRAF6-3 × Flag (0.25 μg). At 48 hpt, cells were fixed with 4% paraformaldehyde (Solarbio, P1110) at RT for 30 min, and then permeabilized with 0.3% Triton X-100 (Beyotime, ST795) at RT for 3 min. After blocking with 5% bovine serum albumin (BSA, Beyotime, ST023) in PBS at RT for 30 min, coverslips were incubated with anti-HA tag (1:300), anti-Myc tag (1:300), or anti-Flag (1:300) tag mAbs as primary antibodies at RT for 1 h, followed by three washes with PBS. Coverslips were then incubated with Alexa Fluor 488-conjugated goat anti-rabbit IgG (H&L) (1:500) or Alexa Fluor 594-conjugated goat anti-mouse IgG (H&L) (1:500) in the dark for 1 h. They were mounted on glass slides using 4’,6-diamidino-2-phenylindole (DAPI) counterstaining solution (Sigma-Aldrich, Merck KGaA, MBD0015). After the mounting medium had solidified, the slides were observed and imaged using a Zeiss LSM510 laser scanning inverted confocal microscope with a 63×/1.4 NA oil immersion objective (Zeiss AG).

For colocalization analysis between PRRSV GP4 or GP5 and endogenous SQSTM1 or TRAF6, Marc-145 cells were infected with PRRSV at a multiplicity of infection (MOI) of 0.1. At 36 hours post-infection (hpi), the samples were fixed, permeabilized, and stained with mouse anti-GP4 mAb (1:300) or anti-GP5 pAb (1:500), together with rabbit anti-SQSTM1 (1:500) or anti-TRAF6 (1:500) pAbs, respectively. The remaining procedures were performed as described above.

### Polyubiquitination assay

To identify the key polyubiquitination site(s) of GP4 and GP5 proteins, the HA-Ub-K63 or His-Ub-K63 and Flag-TRAF6 plasmid were co-transfected into HEK-293 T cells with either pCAGGS-GP4-HA or its mutants (pCAGGS-GP4-K13R-HA, pCAGGS-GP4-K24R-HA, pCAGGS-GP4-K35R-HA, pCAGGS-GP4-K65R-HA, pCAGGS-GP4-K112R-HA, pCAGGS-GP4-K115R-HA, pCAGGS-GP4-K139R-HA), respectively, or pCAGGS-GP5-HA or its mutants (pCAGGS-GP5-K4R-HA, pCAGGS-GP5-K59R-HA, pCAGGS-GP5-K129R-HA, pCAGGS-GP5-K149/163/166/177R-HA), respectively. At 48 hpt, cells were harvested and subjected to co-immunoprecipitation (co-IP) assay using Protein G MagBeads (Glpbio, GK30001) pre-coated with anti-GP4  (1.5 μg per sample) or anti-HA (1.5 μg per sample) mAbs, respectively. The pulled-down products were detected by western blot using the corresponding antibodies.

### Small interfering RNA (siRNA) transfection

Marc-145 cells were seeded into 24-well plates at a density of 1 × 10^5^ cells/mL. Approximately 24 h later, when 80% confluence was reached, the cells were transfected with 100 nM universal negative control siRNA or 25, 50, 100 nM validated specific siRNAs targeting monkey SQSTM1 (siSQSTM1-1, 5ʹ- GCUGUUAAGCUAGUGUAGATT-3ʹ and 5ʹ-UCUACACUAGCUUAACAGCTT-3ʹ), monkey TRAF6 (siTRAF6, 5ʹ-GGUGAAGUGUCCAAAUGAATT-3ʹ and 5ʹ-UUCAUUUGGACACUUCACCTT-3ʹ), or monkey NBR1 (siNBR1, 5ʹ-GGUUGGUAUUUGUAAUGAATT-3ʹ and 5ʹ-UUCAUUACAAAUACCAACCTT-3ʹ) using X-tremeGENE^™^ siRNA Transfection Reagent (Roche Diagnostics, 04476115001). For PAMs, the porcine SQSTM1 siRNA sequence was as follows: 5ʹ-CGAUGACUGGACCCAUUUA-3ʹ and 5ʹ-UAAAUGGGUCCAGUCAUCGTT-3ʹ. At 24 hpt, the cells were harvested to examine mRNA and protein levels of SQSTM1 and TRAF6 via RT-qPCR and western blot, respectively. To evaluate the effects of SQSTM1 knockdown on PRRSV replication, the transfected Marc-145 cells or PAMs were infected with PRRSV at an MOI of 0.1 or 0.01 at 12 hpt and harvested at 36 hpi. The expression of PRRSV N protein and viral titers in the supernatants were measured.

### Statistical analysis

The data were analyzed using GraphPad Prism 8 software (GraphPad Software, Inc., USA). Data are expressed as means ± standard deviations (SD). One-way analysis of variance (ANOVA) was used for comparisons among three or more groups, and the two-tailed unpaired Student’s *t*-test was used for comparisons between two groups. Statistical significance was defined as follows: ^****^P < 0.0001; ^***^P < 0.001; ^**^P < 0.01; ^*^P < 0.05; ns, not significant.

## Results

### Autophagy receptor SQSTM1 inhibits PRRSV replication in vitro

**Figure 1:**
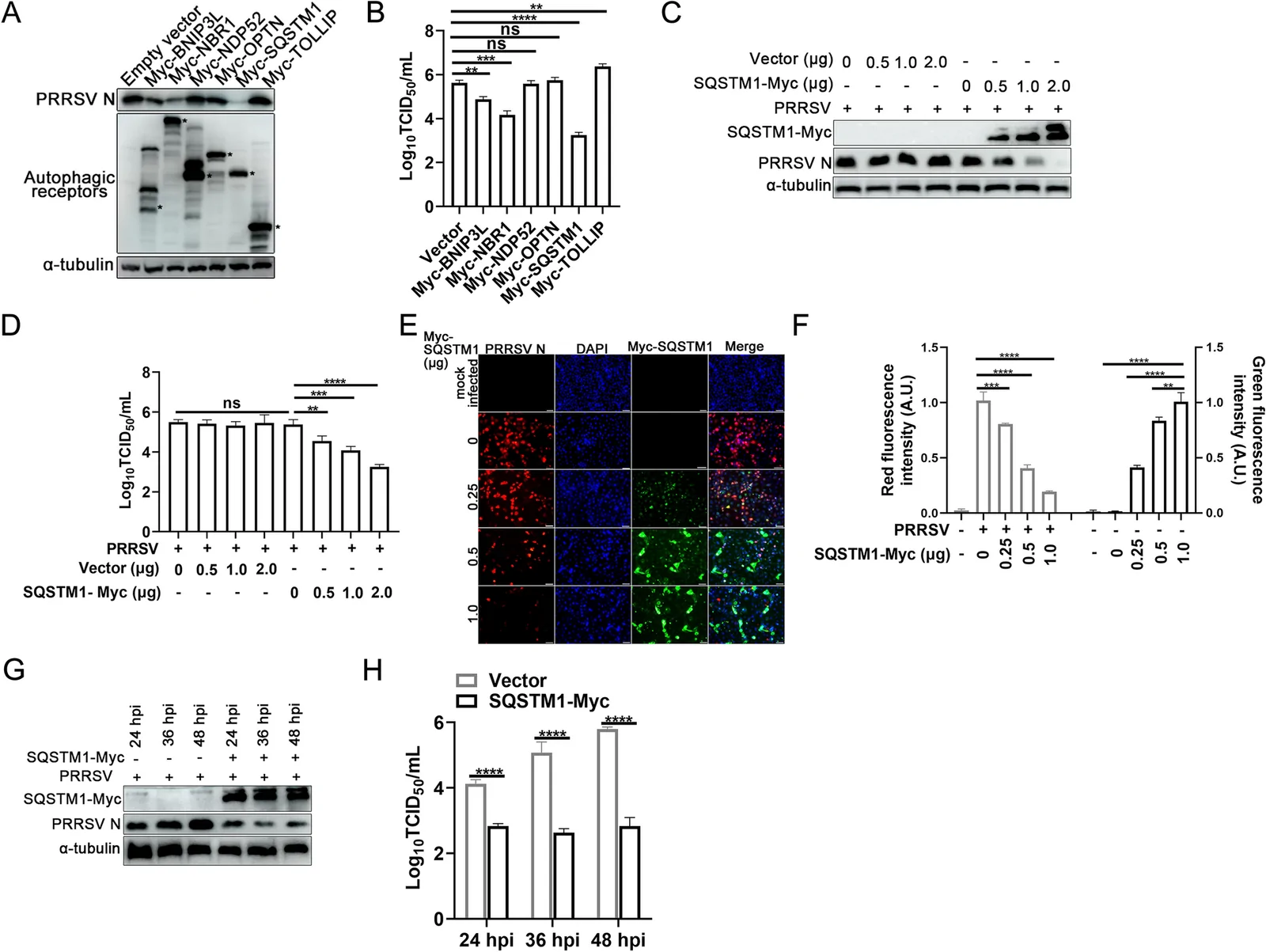
**Overexpression of SQSTM1 inhibits PRRSV replication in vitro. A** and **B** Screening of autophagy receptors for their ability to inhibit PRRSV replication. Marc-145 cells seeded in 12-well plates were separately transfected with 1.0 μg of Myc-BNIP3L, Myc-NBR1, Myc-NDP52, Myc-OPTN, Myc-SQSTM1, or Myc-TOLLIP plasmids. At 12 hpt, cells were inoculated with PRRSV at an MOI of 0.1. At 36 hpi, cells and cell culture supernatants were collected for further analysis. **C** and **D** Effect of SQSTM1 overexpression on PRRSV replication. Marc-145 cells seeded in 6-well plates were transfected with 0, 0.5, 1.0, or 2.0 μg of Myc-SQSTM1 plasmids, followed by inoculation with PRRSV at an MOI of 0.1 at 12 hpt. At 36 hpi, PRRSV N protein abundance was detected by western blot (**C**), and viral titers in supernatants were determined by TCID_50_ assay (**D**). **E** IFA was performed to analyze the effect of SQSTM1 overexpression on PRRSV replication. Marc-145 cells seeded in 12-well plates were transfected with increasing doses of Myc-SQSTM1 (0, 0.25, 0.5, 1.0 μg), followed by inoculation with PRRSV at an MOI of 0.1 at 12 hpt. At 36 hpi, cells were fixed, permeabilized, and incubated with rabbit anti-Myc or mouse anti-N mAbs, along with corresponding fluorescent secondary antibodies; nuclei were stained with DAPI. **F** Quantification of fluorescence intensity shown in (**E**). **G** and **H** Effect of SQSTM1 overexpression on PRRSV replication at different time points. Marc-145 cells seeded in 12-well plates were transfected with 1.0 μg of Myc-SQSTM1 plasmids and infected with PRRSV at an MOI of 0.1 at 12 hpt. At 24, 36, and 48 hpi, cells and supernatants were harvested to detect PRRSV N protein (**G**) and measure supernatant viral titers (**H**), respectively. ^**^P < 0.01, ^***^P < 0.001, ^****^P < 0.0001, ns: not significant. Scale bar: 200 μm.

To investigate whether canonical autophagy receptors play a critical role in host resistance against PRRSV infection, we first transfected Marc-145 cells with eukaryotic expression plasmids (Myc-BNIP3L, Myc-NBR1, Myc-NDP52, Myc-OPTN, Myc-SQSTM1, and Myc-TOLLIP) and subsequently infected the cells with PRRSV at an MOI of 0.1. Western blot results showed that compared with the control group, overexpression of NBR1 (lane 3) and SQSTM1 (lane 6)—especially SQSTM1—markedly suppressed PRRSV replication, as evidenced by reduced expression levels of the PRRSV N protein and decreased viral titers in the supernatant (Figure 1A and B). Overexpression of BNIP3L moderately reduced the expression of PRRSV N protein and viral titers in the supernatant compared with the control group (Figure 1A, lane 2; Figure 1B). In contrast, overexpression of NDP52 or OPTN exerted no apparent effect on PRRSV replication compared with the control group (Figure 1A, lanes 4 and 5; Figure 1B), whereas overexpression of TOLLIP promoted viral replication (Figure 1A, lane 7; Figure 1B). Therefore, the autophagy receptor SQSTM1 was selected to further investigate the regulatory mechanism of selective autophagy in PRRSV replication.

To further corroborate the inhibitory effect of SQSTM1 on PRRSV replication, Marc-145 cells were transfected with increasing doses of Myc-SQSTM1 plasmids and subsequently infected with PRRSV at an MOI of 0.1. The results showed that overexpression of Myc-SQSTM1 in Marc-145 cells concentration-dependently suppressed PRRSV N protein expression (Figure 1C). TCID_50_ analysis of progeny viral titers in the supernatant revealed that, compared with the control group, overexpression of Myc-SQSTM1 reduced viral titers by 0.79, 1.25, and 2.21 log_10_ at the three tested doses, respectively (Figure 1D). Indirect immunofluorescence assay (IFA) was performed to examine the effect of SQSTM1 overexpression on PRRSV N protein expression. As shown in Figure 1E and F, with increasing fluorescence intensity of SQSTM1 (green), the fluorescence intensity of N protein (red) gradually decreased. We also determined whether SQSTM1 inhibits PRRSV replication at different time points. Marc-145 cells were transfected with empty vector or Myc-SQSTM1 plasmids and then infected with PRRSV at an MOI of 0.1. N protein expression and viral titers were analyzed at 24, 36, and 48 hpi. Compared with the control group, overexpression of Myc-SQSTM1 inhibited PRRSV N protein expression at 24, 36, and 48 hpi (Figure 1G). Consistent with this, viral titers decreased by 1.29 log_10_, 2.45 log_10_, and 2.96 log_10_ at these time points, respectively, as determined by TCID_50_ assay (Figure 1H).

### Knockdown of endogenous SQSTM1 promotes PRRSV replication

**Figure 2:**
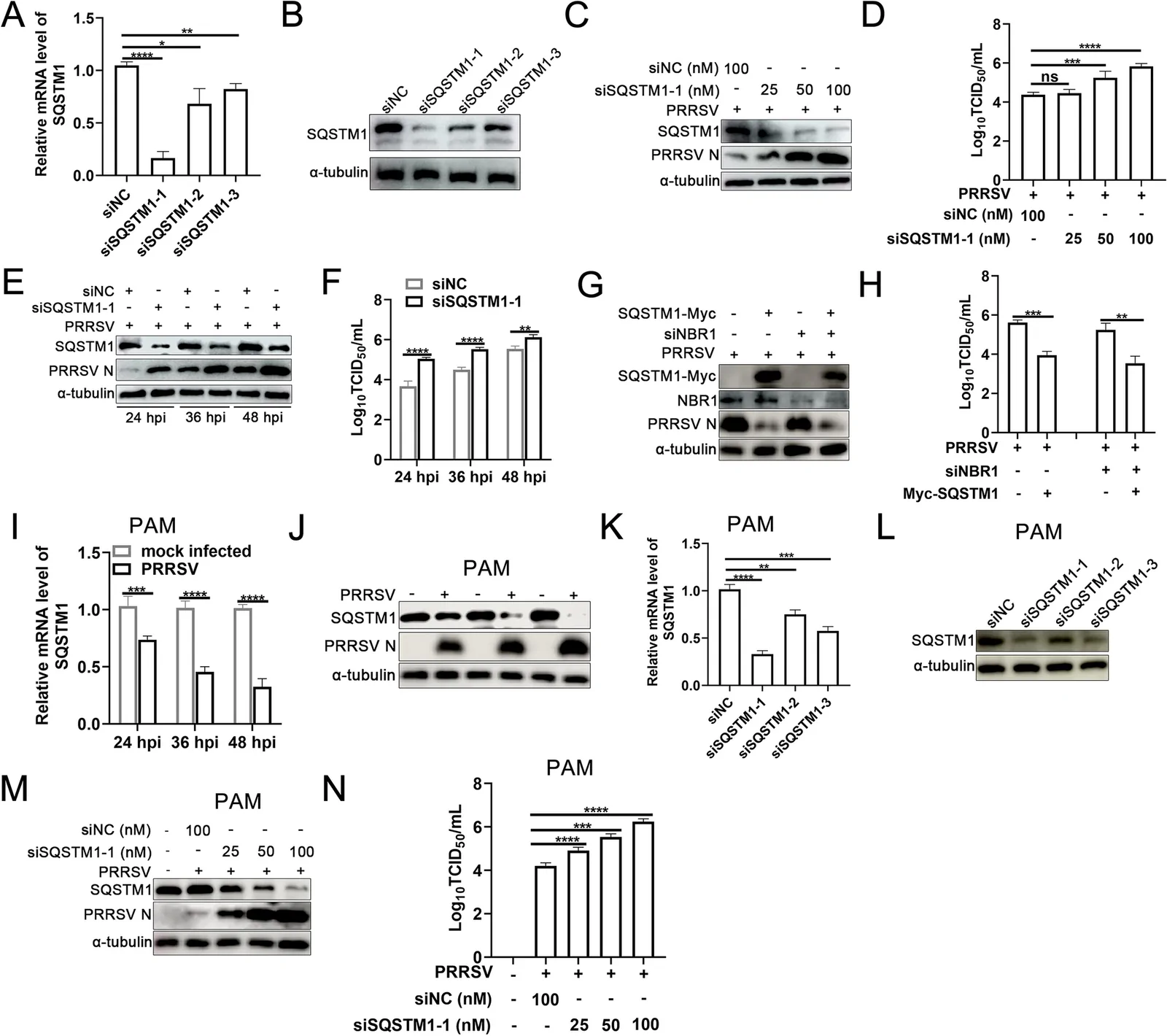
**Knockdown of SQSTM1 promotes PRRSV replication in vitro.** **A** and **B** Validation of the knockdown efficiency of SQSTM1-specific siRNAs. Marc-145 cells were transfected with 100 nM siNC, siSQSTM1-1, siSQSTM1-2, or siSQSTM1-3. At 48 hpt, cells were harvested to detect the mRNA (**A**) and protein (**B**) expression levels of endogenous SQSTM1. **C** and **D** Effect of endogenous SQSTM1 knockdown on PRRSV replication. Marc-145 cells were transfected with 100 nM siNC or gradient concentrations (25, 50, 100 nM) of siSQSTM1-1. Cells were infected with PRRSV at an MOI of 0.1 at 12 hpt. At 36 hpi, cells and supernatants were collected to detect PRRSV N protein expression (**C**) and determine supernatant viral titers (**D**). **E** and **F** Effect of endogenous SQSTM1 knockdown on PRRSV replication at different time points. **G** and **H** Effect of NBR1 knockdown on the SQSTM1-mediated anti-PRRSV activity. **I** and **J** Effect of PRRSV infection on endogenous SQSTM1 expression in PAMs. **K** and **L** RT-qPCR and western blot verification of the knockdown efficiency of different siRNAs targeting porcine SQSTM1. **M** and **N** Effect of endogenous SQSTM1 knockdown on PRRSV replication in PAMs. PAMs cultured in 24-well plates were transfected with siNC or siSQSTM1 targeting porcine SQSTM1. At 12 hpt, cells were infected with PRRSV at an MOI of 0.01. At 36 hpi, cells and supernatants were harvested for detection of PRRSV N protein (**M**) and quantification of viral titers (**N**). ^*^P < 0.05, ^**^P < 0.01, ^***^P < 0.001, ^****^P < 0.0001, ns = not significant.

Next, we further investigated the effect of SQSTM1 knockdown on PRRSV replication. First, the knockdown efficiency of different siRNA targeting SQSTM1 (siSQSTM1) was evaluated. RT-qPCR and western blot results confirmed that, compared with the siNC-transfected control group, siSQSTM1-1 effectively reduced the expression of endogenous SQSTM1 at both mRNA and protein levels (Figure 2A and B). Then siNC or various doses of siSQSTM1-1 were transfected into Marc-145 cells, followed by infection with PRRSV at an MOI of 0.1. Viral infection experiments revealed that, compared with the control group, knockdown of endogenous SQSTM1 enhanced PRRSV N protein expression in a concentration-dependent manner in Marc-145 cells (Figure 2C). TCID_50_ assay of viral titers in the supernatant showed that knockdown of SQSTM1 increased viral titers by 0.08 log_10_, 0.87 log_10_, and 1.45 log_10_, respectively, compared with the control group (Figure 2D).

We further analyzed whether knockdown of SQSTM1 can persistently promote PRRSV replication. Compared with the siNC-transfected group, transfection with 100 nM siSQSTM1-1 evidently increased PRRSV N protein expression at 24, 36, and 48 hpi in Marc-145 cells (Figure 2E). Consistently, supernatant viral titers increased by 1.3 log_10_, 0.9 log_10_, and 0.7 log_10_, respectively, compared with the control group (Figure 2F). Previous studies have demonstrated that SQSTM1 and NBR1 can form heterooligomers via their PB1 domains to cooperatively mediate selective autophagy of ubiquitinated protein aggregates [48]. As shown in Figure 1A, both NBR1 and SQSTM1 suppressed PRRSV replication. Accordingly, we investigated whether NBR1 modulated the anti-PRRSV activity of SQSTM1. As shown in Figure 2G and H, knockdown of endogenous NBR1 failed to abrogate SQSTM1-mediated suppression of PRRSV replication. These data suggest that the two proteins exert their biological functions independently in regulating PRRSV replication.

Additionally, we examined the effect of endogenous SQSTM1 knockdown on PRRSV replication in porcine alveolar macrophages (PAMs), the natural host cells of PRRSV. First, we determined whether PRRSV infection affected endogenous porcine SQSTM1 expression. As shown in Figure 2I and J, PRRSV infection downregulated endogenous SQSTM1 mRNA and protein expression at the indicated time points in PAMs. Next, we validated the knockdown efficiency of the synthesized siRNA specific to porcine SQSTM1. siSQSTM1-1 exhibited the highest knockdown efficiency and was therefore selected for subsequent experiments (Figure 2K and L). Viral infection assay results revealed that compared with the control group, knockdown of endogenous SQSTM1 in PAMs dose-dependently promoted PRRSV N protein expression (Figure 2M), and viral titers in the supernatant increased by 0.71 log_10_, 1.34 log_10_, and 2.05 log_10_, respectively (Figure 2N). These results suggest that SQSTM1 acts as a host restriction factor during PRRSV replication in both Marc-145 cells and PAMs.

### SQSTM1 is involved in the selective autophagic degradation of PRRSV Nsp5, Nsp12, GP4 and GP5 proteins

**Figure 3:**
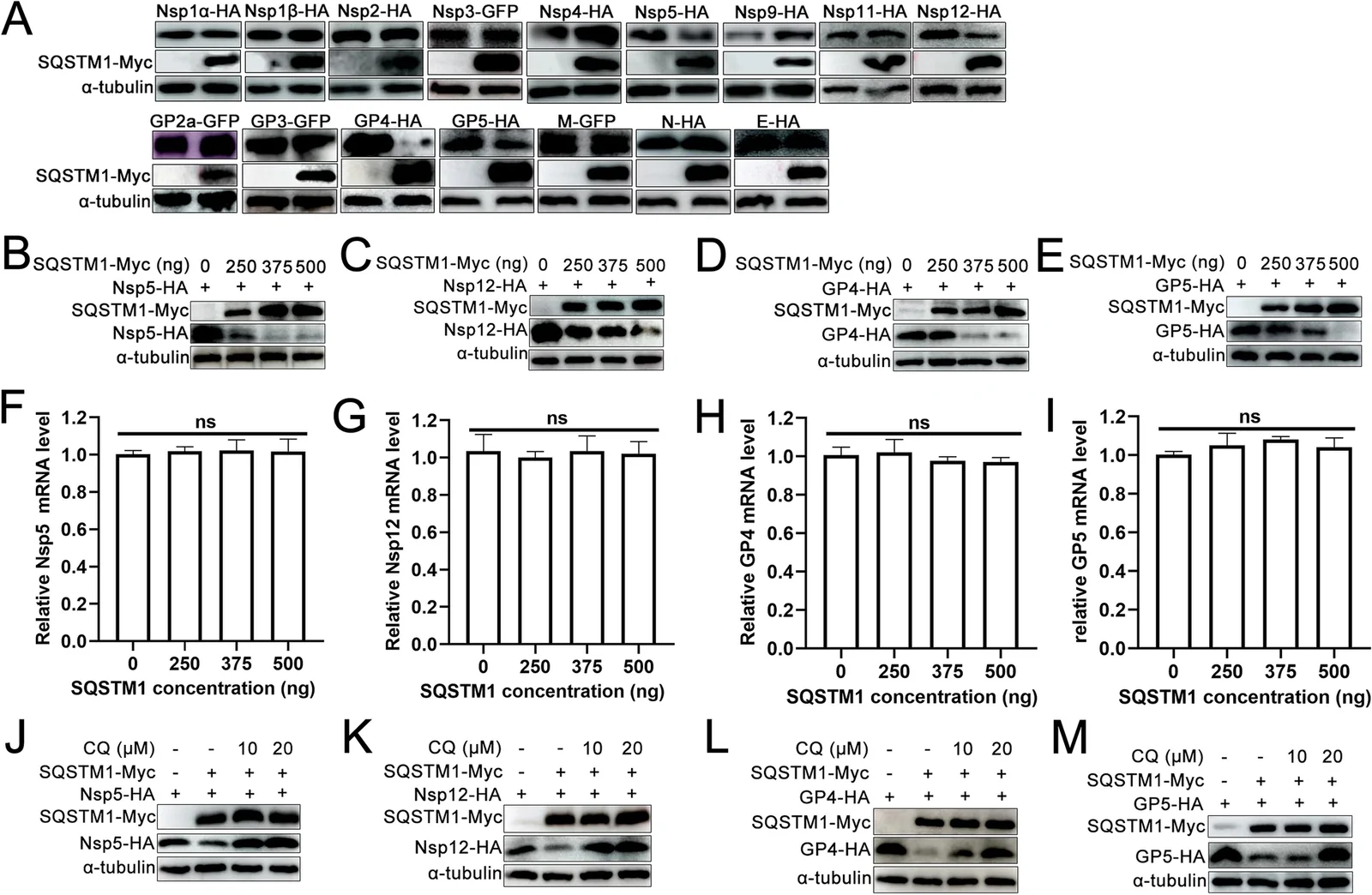
**SQSTM1 mediates the autophagic degradation of PRRSV Nsp5, Nsp12, GP4 and GP5 proteins. A** Screening of PRRSV proteins degraded by SQSTM1. Marc-145 cells seeded in 12-well plates were co-transfected with 0.5 μg of empty vector or Myc-SQSTM1 plasmid, together with 0.5 μg of HA-tagged or GFP-tagged plasmids expressing PRRSV non-structural or structural proteins. At 48 hpt, cells were collected to detect the expression of viral proteins. **B**–**E** Dose-dependent degradation of PRRSV Nsp5, Nsp12, GP4, and GP5 proteins by SQSTM1. Marc-145 cells were co-transfected with 0, 0.25, 0.375, and 0.5 μg of Myc-SQSTM1 plasmid combined with 0.5 μg of HA-Nsp5, HA-Nsp12, HA-GP4, or HA-GP5 plasmid, respectively. At 36 hpt, cells were collected and the protein expression levels of HA-Nsp5 (**B**), HA-Nsp12 (**C**), HA-GP4 (**D**), and HA-GP5 (**E**) were determined by western blot. The corresponding mRNA expression levels were quantified by RT-qPCR (**F**–**I**). **J**–**M** Effect of the autophagic inhibitor CQ on SQSTM1-mediated degradation of Nsp5, Nsp12, GP4 and GP5. Marc-145 cells seeded in 12-well plates were co-transfected with 0.5 μg of Myc-SQSTM1 and 0.5 μg of individual HA-tagged viral protein plasmids. At 24 hpt, transfected cells were treated with DMSO or increasing concentrations of CQ (10 μM and 20 μM) for an additional 12 h. Cells were collected at 36 hpt, and the protein expression of HA-Nsp5 (**J**), HA-Nsp12 (**K**), HA-GP4 (**L**), and HA-GP5 (**M**) was analyzed by western blot. ns: not significant.

Accumulating evidence suggests that autophagy cargo receptors play a critical role in delivering viral components to autophagosomes for selective degradation [49]. Therefore, we determined whether autophagy cargo receptor SQSTM1 participates in degrading PRRSV protein(s) to suppress viral replication. We co-transfected Marc-145 cells with Myc-SQSTM1 and HA- or GFP-tagged plasmids expressing PRRSV non-structural or structural proteins. Western blot results showed that among all detected viral proteins, overexpression of SQSTM1 promoted the degradation of HA-Nsp5, HA-Nsp12, HA-GP4, and HA-GP5, while causing no obvious degradation on other viral proteins (Figure 3A). We thus further confirmed whether SQSTM1 is involved in the degradation of PRRSV Nsp5, Nsp12, GP4, or GP5 proteins. Marc-145 cells were co-transfected with increasing doses of Myc-SQSTM1 plasmids together with HA-Nsp5, HA-Nsp12, HA-GP4, or HA-GP5, and the mRNA and protein expression levels of these proteins were detected. Western blot results revealed that SQSTM1 induced dose-dependent degradation of HA-Nsp5 (Figure 3B), HA-Nsp12 (Figure 3C), HA-GP4 (Figure 3D), and HA-GP5 (Figure 3E), without affecting their mRNA levels (Figure 3F–I).

To verify that SQSTM1 mediates the autophagic degradation of PRRSV Nsp5, Nsp12, GP4, and GP5 proteins, Marc-145 cells were co-transfected with Myc-SQSTM1 and HA-Nsp5, HA-Nsp12, HA-GP4, or HA-GP5 plasmids, followed by treatment with increasing doses of CQ. CQ treatment dose-dependently reversed SQSTM1 overexpression-induced degradation of HA-Nsp5 (Figure 3J), HA-Nsp12 (Figure 3K), HA-GP4 (Figure 3L), and HA-GP5 (Figure 3M). Given that GP4 and GP5 are key envelope proteins that play critical roles in PRRSV entry into host cells and virion assembly, we selected GP4 and GP5 for subsequent mechanistic investigation of SQSTM1-mediated degradation of these viral proteins.

### SQSTM1 interacts with PRRSV GP4 and GP5 proteins and promotes the autophagy-lysosomal degradation of GP4

**Figure 4:**
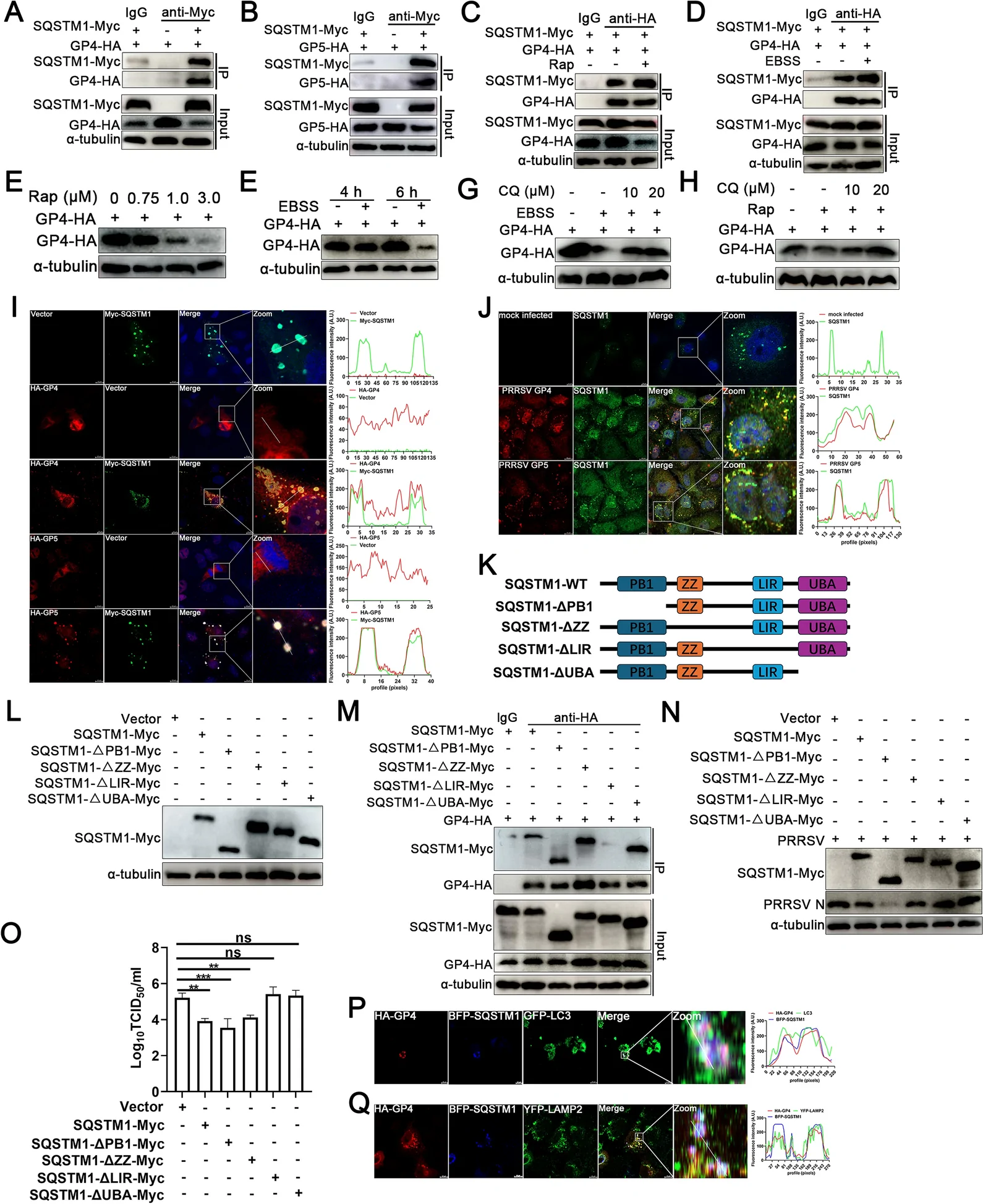
**SQSTM1 interacts with PRRSV GP4 and GP5 and targets GP4 for autophagy–lysosomal degradation. A** and **B** Interaction between SQSTM1 and GP4 or GP5. HEK-293 T cells were co-transfected with Myc-SQSTM1 and HA-GP4 or HA-GP5. At 48 hpt, cell lysates were subjected to a co-IP assay. **C** and **D** Activation of autophagy (Rap or EBSS starvation) enhances the SQSTM1–GP4 association. **E** and **F** Effects of autophagy activation on GP4 degradation. Marc-145 cells were transfected with the HA-GP4 plasmid and subsequently treated with gradient concentrations of Rap (0, 0.75, 1.0, and 3.0 μM), starved in EBSS, or maintained in normal 10% FBS DMEM for 4 or 6 h, followed by western blot analysis of HA-GP4. **G** and **H** CQ blocks Rap- or EBSS-induced GP4 degradation in Marc-145 cells. **I** Colocalization analysis of HA-GP4 or HA-GP5 with Myc-SQSTM1. **J** Colocalization of endogenous SQSTM1 with PRRSV GP4 or GP5 during viral infection. **K** and **L** Schematic and western blot validation of Myc-tagged SQSTM1 truncation mutants lacking the PB1, ZZ, UBA, or LIR domain. **M** Co-IP mapping the GP4-binding region of SQSTM1. **N** and **O** Effects of SQSTM1 truncation mutants on PRRSV replication. Marc-145 cells were transfected with individual SQSTM1 truncation mutant plasmids. At 12 hpt, cells were infected with PRRSV at an MOI of 0.1. Cells and cell culture supernatants were harvested at 36 hpi to detect N protein expression (**N**) and quantify viral titers in supernatants (**O**). **P** and **Q** SQSTM1 delivers GP4 to autophagosomes and lysosomal compartments for degradation. HEK-293 T cells were co-transfected with empty vector or BFP-SQSTM1, along with HA-GP4 and GFP-LC3 or YFP-LAMP2. At 48 hpt, cells were fixed and examined by confocal microscopy. ^**^P < 0.01, ^***^P < 0.001, *ns* not significant. Scale bar: 10 μm.

Since SQSTM1 can mediate degradation of PRRSV GP4 and GP5, we further examined whether SQSTM1 interacts with GP4 or GP5. HEK-293 T cells were co-transfected with Myc-SQSTM1 and HA-GP4 or HA-GP5 plasmids, followed by co-IP using Protein G MagBeads pre-coated with anti-Myc tag mAb. The results showed that Myc-SQSTM1 effectively pulled down HA-GP4, as illustrated in Figure 4A. Similarly, Myc-SQSTM1 also effectively pulled down HA-GP5 (Figure 4B). In addition, induction of autophagic flux by Rap or EBSS treatment enhanced the interaction between HA-GP4 and Myc-SQSTM1 (Figure 4C and D). Moreover, Rap or EBSS treatment effectively promoted GP4 protein degradation in a concentration‑dependent and time‑dependent manner, respectively (Figure 4E and F). In contrast, inhibition of autophagy with CQ reversed the Rap- or EBSS-induced degradation of GP4 in a concentration-dependent manner (Figure 4G and H).

Confocal microscopy was performed to assess the subcellular localization of HA-GP4 or HA-GP5 and Myc-SQSTM1. The results indicated that both HA-GP4 and HA-GP5 readily colocalized with Myc-SQSTM1 and displayed a punctate distribution (Figure 4I). We also investigated the subcellular localization of GP4 or GP5 and endogenous SQSTM1 during PRRSV infection. Confocal microscopy revealed that PRRSV GP4 and GP5 proteins readily colocalized with endogenous SQSTM1 upon viral infection (Figure 4J).

It has been demonstrated that the PB1, UBA, and LIR domains of SQSTM1 play critical roles in mediating oligomerization, binding to ubiquitinated substrates, and interacting with LC3, respectively [50]. We thus further mapped the domain(s) responsible for the interaction between SQSTM1 and GP4. First, successful expression of each SQSTM1 mutant, including Myc-SQSTM1-ΔPB1, Myc-SQSTM1-ΔZZ, Myc-SQSTM1-ΔUBA, and Myc-SQSTM1-ΔLIR, was verified (Figure 4K and L). Myc-SQSTM1, Myc-SQSTM1-ΔPB1, Myc-SQSTM1-ΔZZ, Myc-SQSTM1-ΔUBA, and Myc-SQSTM1-ΔLIR plasmids were then co-transfected into HEK-293 T cells with HA-GP4. Cell samples were subsequently collected for co-IP analysis. The results indicated that except for the Myc-SQSTM1-ΔLIR mutant, all other mutants effectively pulled down HA-GP4 (Figure 4M).

Since SQSTM1 inhibits PRRSV replication by mediating the autophagic degradation of GP4, we further clarified the key domain(s) of SQSTM1 responsible for inhibiting PRRSV replication. Marc-145 cells were transfected with Myc-SQSTM1-ΔPB1, Myc-SQSTM1-ΔZZ, Myc-SQSTM1-ΔUBA, or Myc-SQSTM1-ΔLIR, followed by infection with PRRSV. Transfection with Myc-SQSTM1, Myc-SQSTM1-ΔPB1, or Myc-SQSTM1-ΔZZ suppressed PRRSV N protein expression (Figure 4N) and reduced viral titers in the supernatant (Figure 4O). In contrast, transfection with Myc-SQSTM1-ΔUBA or Myc-SQSTM1-ΔLIR completely failed to inhibit PRRSV N protein expression (Figure 4N) and reduce viral titers in the supernatant (Figure 4O). Collectively, these results indicate that the LIR domain is essential for the interaction between SQSTM1 and GP4, while both the LIR and UBA domains are required for SQSTM1-mediated inhibition of PRRSV replication.

We selected the PRRSV GP4 protein as the target protein to investigate whether SQSTM1 facilitates viral protein degradation through the autophagy–lysosomal pathway. HEK-293 T cells were transfected with HA-GP4 and GFP-LC3, with or without BFP-SQSTM1. The results showed that overexpression of BFP-SQSTM1 led to the formation of prominent cytoplasmic puncta (Figure 4P), indicating that overexpression of SQSTM1 markedly promotes trafficking of PRRSV GP4 to LC3-positive autophagic vesicles. Confocal microscopy further revealed that BFP-SQSTM1 overexpression redirected GP4 to LAMP2-positive lysosomal compartments (Figure 4Q). Taken together, these findings indicate that SQSTM1 promotes the degradation of PRRSV GP4 via the autophagy–lysosomal pathway.

### SQSTM1 promotes PRRSV GP4 and GP5 degradation via recruitment of host E3 ubiquitin ligase TRAF6

**Figure 5:**
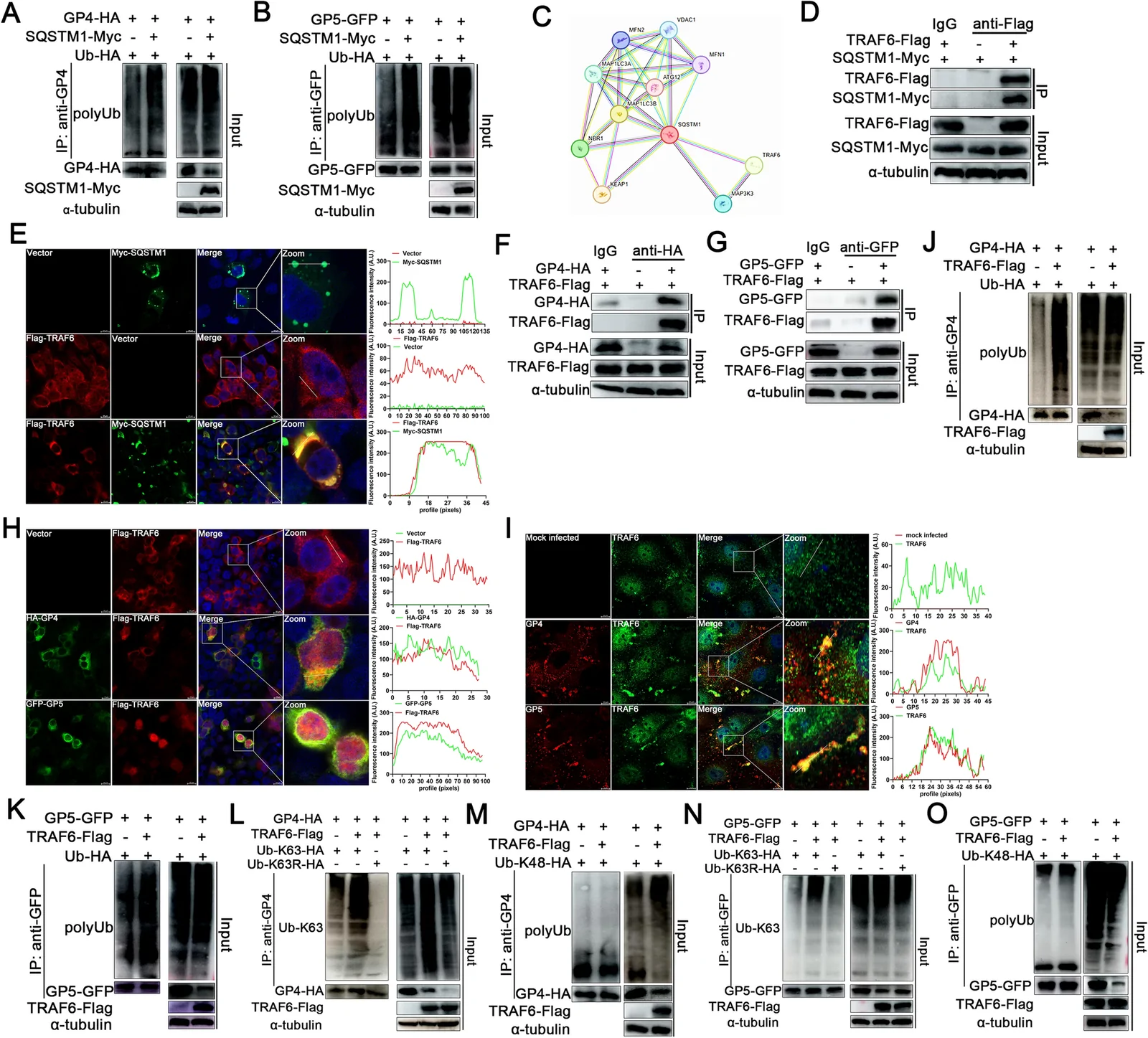
**SQSTM1 recruits the host E3 ubiquitin ligase TRAF6 to facilitate the ubiquitination and degradation of GP4 and GP5. A** Effect of SQSTM1 on GP4 ubiquitination. Empty vector or Myc-SQSTM1 was co-transfected into HEK-293 T cells, together with HA-GP4 and HA-Ub. At 48 hpt, cells were collected and ubiquitination levels of GP4 were detected via co-IP assay. **B** Effect of SQSTM1 on GP5 ubiquitination. **C** Screening of porcine SQSTM1-interacting E3 ubiquitin ligases using the online protein interaction database STRING. **D** SQSTM1 interacts with TRAF6. HEK-293 T cells were co-transfected with Flag-TRAF6 and Myc-SQSTM1. At 48 hpt, cell lysates were subjected to co-IP assay. **E** Confocal analysis of the subcellular colocalization of TRAF6 and SQSTM1. Marc-145 cells were transfected with Flag-TRAF6 alone, Myc-SQSTM1 alone, or co-transfected with both plasmids. At 36 hpt, cells were fixed, permeabilized, and incubated with a mouse anti-Myc mAb or a rabbit anti-Flag mAb, followed by incubation with the corresponding fluorescent secondary antibodies. **F** Co-IP assay was performed to determine whether GP4 interacts with TRAF6. HEK-293 T cells were co-transfected with Flag-TRAF6 and HA-GP4. At 48 hpt, cells were collected for co-IP assay using beads pre-coated with anti-HA mAb. **G** Co-IP was performed to determine whether GP5 interacts with TRAF6. **H** Confocal detection of the subcellular distribution of HA-GP4, GFP-GP5, and Flag-TRAF6. **I** Subcellular localization of GP4, GP5 and TRAF6 during PRRSV infection. Marc-145 cells infected with PRRSV at an MOI of 0.1 were fixed, permeabilized, and incubated with mouse anti-GP4 mAb, mouse anti-GP5 pAb, and rabbit anti-TRAF6 pAb, followed by incubation with the corresponding fluorescent secondary antibodies. **J** and **K** Effects of TRAF6 on the ubiquitination of GP4 and GP5. **L** and **M** Effects of TRAF6 on K63-linked (**L**) and K48-linked (**M**) ubiquitination of GP4. **N** and **O** Effects of TRAF6 on K63-linked (**N**) and K48-linked (**O**) ubiquitination of GP5. Scale bar: 10 μm.

Given that the ubiquitin binding of SQSTM1 is indispensable for SQSTM1-dependent substrate degradation, we hypothesized that PRRSV GP4 and GP5 proteins undergo polyubiquitination during SQSTM1-induced autophagic degradation. To test this hypothesis, we co-transfected HEK-293 T cells with HA-Ub, Myc-SQSTM1, and either HA-GP4 or GFP-GP5 plasmids to assess the effect of SQSTM1 on polyubiquitination of GP4 and GP5. Co-IP results showed that overexpression of SQSTM1 enhanced polyubiquitination of both GP4 and GP5 proteins (Figure 5A and B). Since SQSTM1 itself lacks E3 ubiquitin ligase activity, we sought to identify the E3 ubiquitin ligase recruited by SQSTM1 that mediates polyubiquitination of GP4 and GP5. In silico protein–protein interaction predictions from the online tool STRING database (version 11.5; https://cn.string-db.org/) suggested a potential interaction between porcine SQSTM1 and the host E3 ubiquitin ligase TRAF6 (Figure 5C). Subsequently, a co-IP assay was performed to verify the interaction between SQSTM1 and TRAF6. The results confirmed that Flag-TRAF6 specifically interacted with Myc-SQSTM1 (Figure 5D). Additionally, confocal microscopy showed that Flag-TRAF6 exhibited evident colocalization with Myc-SQSTM1 (Figure 5E). We next examined whether TRAF6 interacts with PRRSV GP4 or GP5. HEK-293 T cells were co-transfected with Flag-TRAF6 and either HA-GP4 or HA-GP5, followed by a co-IP assay. As expected, both HA-GP4 (Figure 5F) and HA-GP5 (Figure 5G) efficiently pulled down Flag-TRAF6. Furthermore, confocal results showed that Flag-TRAF6 colocalized with HA-GP4 and HA-GP5 (Figure 5H). During viral infection, Flag-TRAF6 also partially colocalized with PRRSV GP4 or GP5 (Figure 5I).

We speculated that TRAF6 might catalyze the polyubiquitination of GP4 or GP5 proteins, and that ubiquitinated GP4 or GP5 would then be degraded upon binding to the autophagy receptor SQSTM1. To verify this hypothesis, we first determined whether TRAF6 mediates polyubiquitination of GP4 or GP5. Co-IP results showed that overexpression of Flag-TRAF6 robustly catalyzed the polyubiquitination of HA-GP4 (Figure 5J). Similarly, TRAF6-catalyzed polyubiquitination of GFP-GP5 was also observed (Figure 5K). Further analysis revealed that TRAF6 catalyzed the K63-linked polyubiquitination of HA-GP4 (Figure 5L), but had no effect on its K48-linked polyubiquitination (Figure 5M). For GP5, TRAF6 catalyzed K63-linked rather than K48-linked polyubiquitination of GFP-GP5 (Figure 5N and O), indicating that TRAF6 preferentially mediates K63-linked polyubiquitination of GP4 and GP5 proteins.

### The polyubiquitination of GP4 at K115 and GP5 at K59 is required for SQSTM1-mediated autophagic degradation

**Figure 6:**
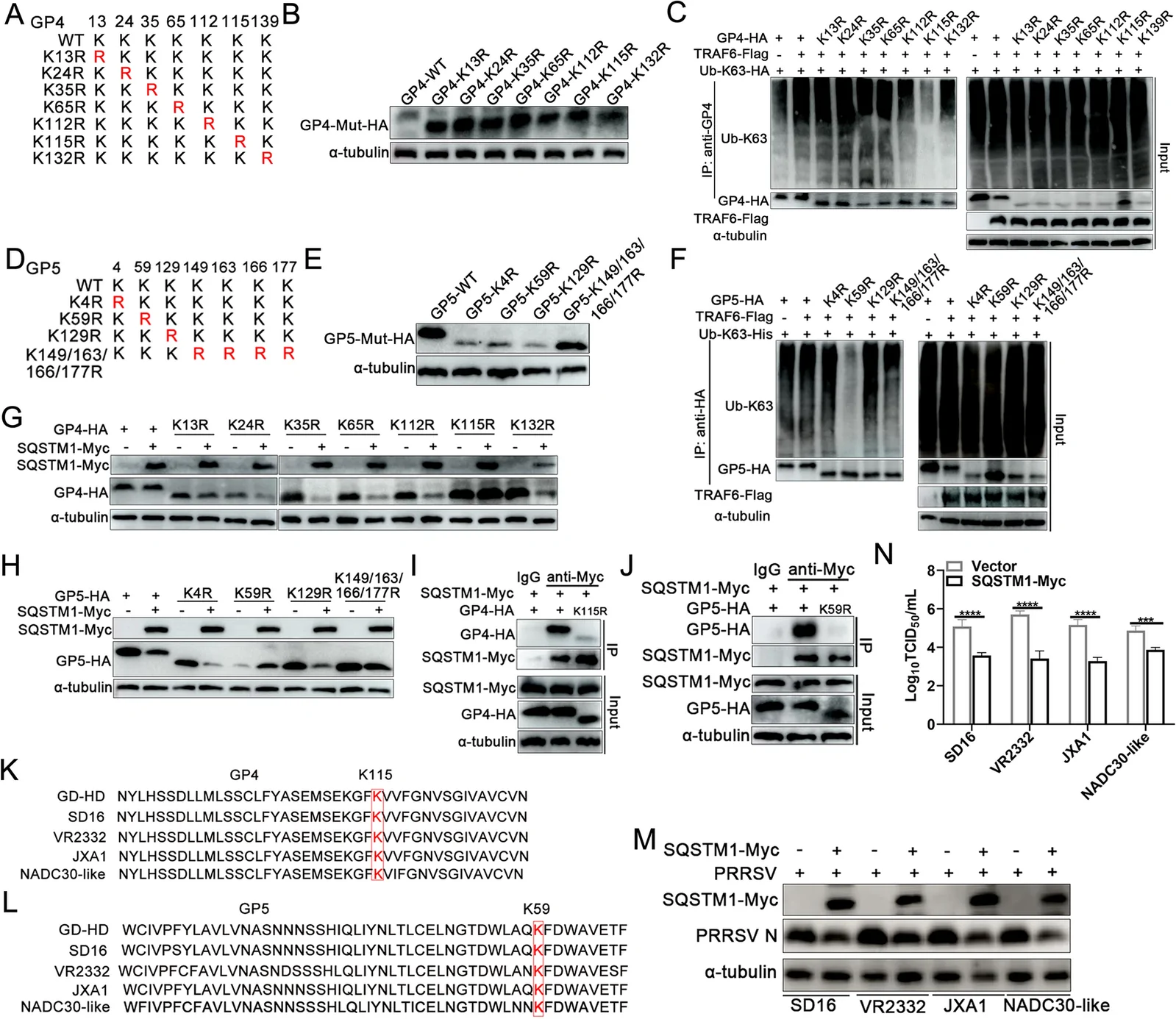
**Ubiquitination of GP4 at K115 residue and GP5 at K59 residue facilitates SQSTM1-mediated degradation of GP4 and GP5. A** Schematic diagram of various lysine (K) point mutants of GD-HD PRRSV GP4 (red ‘R’). **B** Western blot verification of the protein expression of GP4 mutants in HEK-293 T cells. **C** Identification of TRAF6-targeted ubiquitination sites on GP4 via co-IP assay. HEK-293 T cells were co-transfected with HA-GP4 (wild-type or individual mutant), Flag-TRAF6, and HA-Ub plasmids. At 48 hpt, cells were harvested and subjected to co-IP assay using beads pre-coated with anti-GP4 mAb. **D** Schematic diagram of various lysine (K) point mutants of GD-HD PRRSV GP5 (red ‘R’). **E** Western blot verification of the expression of GP5 mutants in HEK-293 T cells. **F** The K site(s) of GP5 catalyzed for ubiquitination by TRAF6 were identified. **G** Effects of SQSTM1 on the degradation of wild-type GP4 and GP4 mutants. Marc-145 cells were co-transfected with empty vector or Myc-SQSTM1 together with HA-GP4-WT or individual HA-GP4 mutant plasmids. At 48 hpt, cells were harvested, and western blot was performed to detect the expression levels of wild-type and mutant GP4 proteins. **H** Identification of GP5 mutants resistant to SQSTM1-mediated degradation. **I** and **J** A co-IP assay was performed to determine whether GP4-K115R (**I**), or GP5-K59R (**J**) interacts with SQSTM1. **K** and **L** Amino acid sequence alignment and analysis of GP4 and GP5 among different PRRSV strains. **M** and **N** Effect of SQSTM1 overexpression on the replication of different PRRSV strains. Marc-145 cells were transfected with 1.0 μg of Myc-SQSTM1 plasmids. At 12 hpt, cells were inoculated with PRRSV strains (SD16, VR2332, JXA1, or NADC30-like) at an MOI of 0.1. Cells and supernatants were harvested at 36 hpi for detection of PRRSV N protein expression (**M**) and quantification of supernatant viral titers (**N**). ^***^P < 0.001, ^****^P < 0.0001, ns: not significant.

Given that polyubiquitination of substrate proteins typically occurs at lysine (K) residues, we further determined which K residue(s) of GP4 are ubiquitinated by TRAF6. A series of GP4 mutant plasmids, in which individual K residues were mutated to arginine (R), were constructed and designated as HA-GP4-K13R, HA-GP4-K24R, HA-GP4-K35R, HA-GP4-K65R, HA-GP4-K112R, HA-GP4-K115R, and HA-GP4-K139R (Figure 6A). These mutant plasmids were transfected into HEK-293 T cells to verify their expression (Figure 6B). The mutants were then co-transfected into HEK-293 T cells together with HA-Ub-K63 and Flag-TRAF6 plasmids for polyubiquitination assays. The results showed that none of the mutations affected the K63-linked polyubiquitination of GP4, with the exception of the HA-GP4-K115R mutant (Figure 6C), indicating that the K115 residue is critical for TRAF6-catalyzed K63-linked polyubiquitination of GP4. Similarly, a series of GP5 mutant plasmids were constructed to examine TRAF6-mediated K63-linked polyubiquitination (Figure 6D and E). TRAF6 efficiently promoted K63-linked polyubiquitination of HA-GP5, HA-GP5-K4R, HA-GP5-K129R, and HA-GP5-K149/163/166/177R, with the exception of HA-GP5-K59R (Figure 6F), suggesting that the K59 residue of GP5 is the major site for TRAF6-catalyzed K63-linked polyubiquitination.

To investigate the role of the K115 residue of GP4 in SQSTM1-mediated autophagic degradation, Marc-145 cells were co-transfected with Myc-SQSTM1 and HA-GP4, HA-GP4-K13R, HA-GP4-K24R, HA-GP4-K35R, HA-GP4-K65R, HA-GP4-K112R, HA-GP4-K115R, or HA-GP4-K139R, followed by detection of HA-GP4 and its mutant protein levels. Compared with the control group, overexpression of SQSTM1 led to the degradation of HA-GP4, HA-GP4-K13R, HA-GP4-K24R, HA-GP4-K35R, HA-GP4-K65R, HA-GP4-K112R, and HA-GP4-K139R, whereas this degradative effect was not observed for the HA-GP4-K115R mutant (Figure 6G). These results indicate that the K115 residue of GP4 is the key site for SQSTM1-mediated autophagic degradation of GP4. Similarly, we analyzed the role of the K59 residue in SQSTM1-mediated autophagic degradation of GP5. As expected, overexpression of SQSTM1 triggered degradation of HA-GP5, HA-GP5-K4R, HA-GP5-K129R, and HA-GP5-K149/163/166/177R, but not HA-GP5-K59R, indicating that the K59 residue is critical for SQSTM1-induced degradation of GP5 (Figure 6H).

Co-IP assays were performed to investigate whether the ubiquitination of K115 residue plays a critical role in mediating the interaction between SQSTM1 and GP4. As shown in Figure 6I, Myc-SQSTM1 effectively pulled down HA-GP4, but not HA-GP4-K115R. Similarly, co-IP results demonstrated that Myc-SQSTM1 interacted with HA-GP5, whereas it failed to interact with HA-GP5-K59R (Figure 6J). These results suggest that SQSTM1-mediated degradation of GP4 and GP5 depends on K63-linked polyubiquitination at GP4 K115 and GP5 K59.

Given that GP4 K115 and GP5 K59 are critical for SQSTM1-mediated autophagic degradation, we performed sequence alignment of GP4 and GP5 from different PRRSV strains. The results showed that GP4 K115 and GP5 K59 are highly conserved among the GD‑HD, SD16, VR2332, JXA1, and NADC30-like PRRSV strains (Figure 6K and L). Based on these findings, we further verified the effect of SQSTM1 on the replication of these PRRSV strains. As shown in Figure 6M, overexpression of SQSTM1 effectively suppressed N protein expression of the SD16, VR2332, JXA1, and NADC30-like strains. TCID_50_ measurement of viral titers in the supernatant revealed that overexpression of SQSTM1 reduced the titers of SD16, VR2332, JXA1, and NADC30-like strains by 1.5 log_10_, 2.29 log_10_, 1.87 log_10_, and 1.0 log_10_, respectively (Figure 6N).

### SQSTM1 and TRAF6 collaboratively promote the autophagic degradation of PRRSV Nsp5, Nsp12, GP4 and GP5 proteins to suppress viral replication

**Figure 7:**
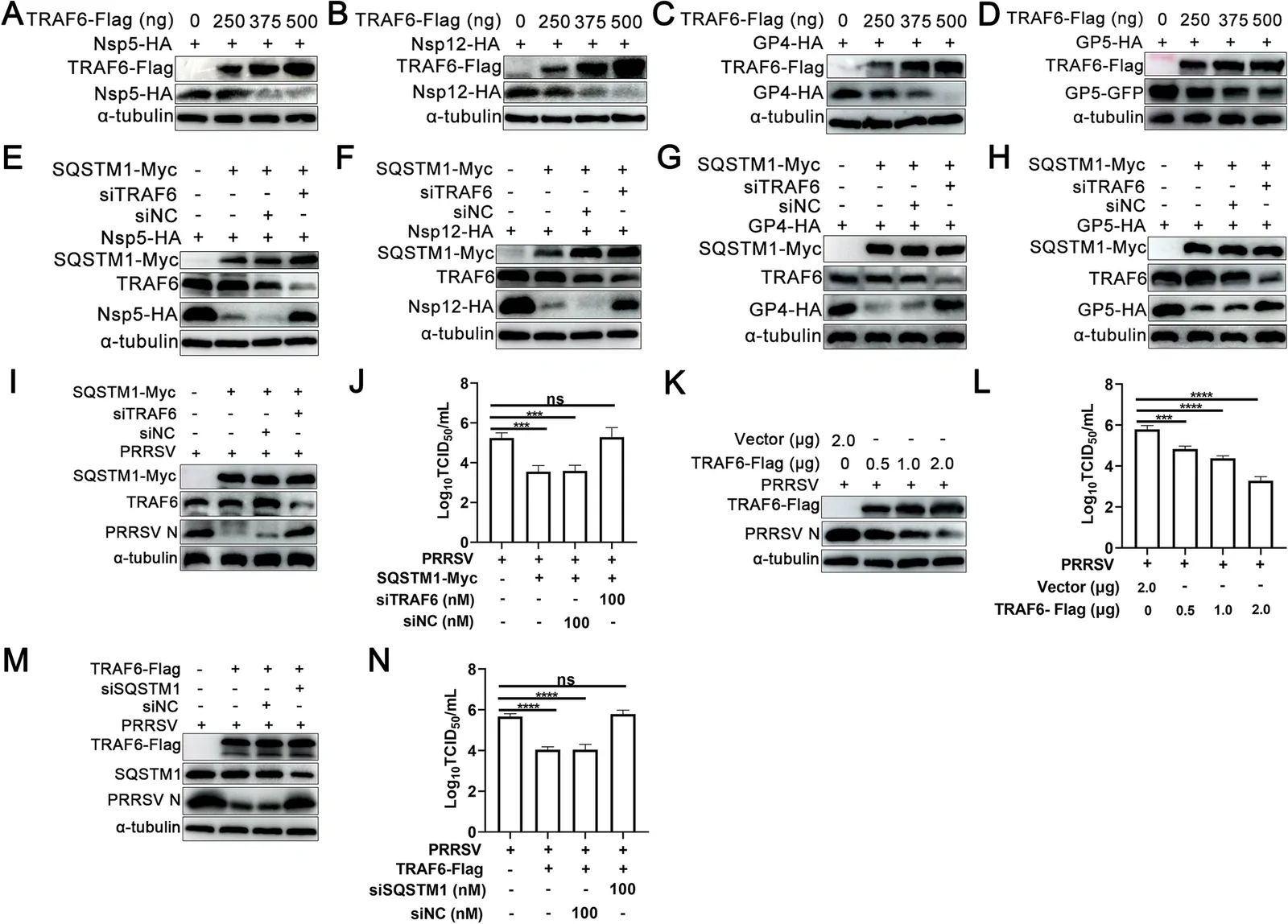
**SQSTM1 and TRAF6 collaboratively promote the autophagic degradation of PRRSV Nsp5, Nsp12, GP4 and GP5 proteins to suppress viral replication. A**–**D** Effect of TRAF6 overexpression on the degradation of PRRSV Nsp5, Nsp12, GP4, and GP5. Marc-145 cells seeded in 12-well plates were co-transfected with gradient doses (0, 0.25, 0.375, and 0.5 μg) of Flag-TRAF6 and 0.5 μg of individual HA-tagged Nsp5, Nsp12, GP4, and GP5 plasmids. At 48 hpt, cells were harvested to determine the protein levels of HA-Nsp5 (**A**), HA-Nsp12 (**B**), HA-GP4 (**C**), and HA-GP5 (**D**). **E**–**H** Effect of TRAF6 knockdown on SQSTM1-mediated degradation of Nsp5, Nsp12, GP4, and GP5. Marc-145 cells were co-transfected with Myc-SQSTM1 and individual HA-tagged viral protein plasmids, along with 100 nM siNC or siTRAF6. At 48 hpt, cells were collected and viral protein expression was analyzed by western blot. **I** and **J** Effect of TRAF6 knockdown on SQSTM1-mediated inhibition of PRRSV replication. Marc-145 cells were transfected with Myc-SQSTM1 and subsequently treated with 100 nM siNC or siTRAF6 at 12 hpt. At 24 hpt, cells were inoculated with PRRSV at an MOI of 0.1. At 36 hpi, cells and cell culture supernatants were collected to detect PRRSV N protein expression (**I**) and quantify viral titers (**J**). **K** and **L** Effect of TRAF6 overexpression on PRRSV replication in Marc-145 cells. **M** and **N** Effect of SQSTM1 knockdown on TRAF6-mediated suppression of PRRSV replication. Marc-145 cells transfected with Flag-TRAF6 were further transfected with 100 nM siNC or siSQSTM1 at 12 hpt and then inoculated with PRRSV at an MOI of 0.1. At 36 hpi, cells and cell culture supernatants were collected to detect PRRSV N protein (**M**) and measure viral titers (**N**). ^***^P < 0.001, ^****^P < 0.0001, ns: not significant.

The effect of TRAF6 on the stability of Nsp5, Nsp12, GP4, or GP5 was assessed. Increasing concentrations of Flag-TRAF6 were co-transfected with HA-Nsp5, HA-Nsp12, HA-GP4, or HA-GP5 plasmids into Marc-145 cells, respectively. Subsequently, protein expression levels of HA-Nsp5, HA-Nsp12, HA-GP4, and HA-GP5 were analyzed. Overexpression of Flag-TRAF6 degraded HA-Nsp5 (Figure 7A), HA-Nsp12 (Figure 7B), HA-GP4 (Figure 7C), and HA-GP5 proteins (Figure 7D) in a dose-dependent manner. We then determined whether TRAF6 participates in SQSTM1-mediated degradation of PRRSV Nsp5, Nsp12, GP4, and GP5 proteins. Overexpression of Myc-SQSTM1 induced degradation of HA-Nsp5, HA-Nsp12, HA-GP4, and HA-GP5 proteins, whereas knockdown of TRAF6 partially reversed SQSTM1 overexpression-mediated degradation of these proteins (Figure 7E–H). These results indicate that SQSTM1 promotes the degradation of PRRSV Nsp5, Nsp12, GP4, and GP5 proteins by recruiting TRAF6.

We next investigated whether TRAF6 plays a critical role in mediating the anti-PRRSV activity of SQSTM1. Marc-145 cells were co-transfected with Myc-SQSTM1 and siNC or siTRAF6, followed by infection with PRRSV at an MOI of 0.1. Overexpression of Myc-SQSTM1 reduced PRRSV N protein expression; in contrast, knockdown of endogenous TRAF6 partially abolished the inhibitory effect of SQSTM1 on PRRSV N protein expression (Figure 7I). TCID_50_ analysis showed that overexpression of SQSTM1 reduced viral titers in the supernatant by 1.71 log_10_, whereas knockdown of TRAF6 in the context of SQSTM1 overexpression increased viral titers by 1.71 log_10_ compared with the siNC group (Figure 7J).

**Figure 8:**
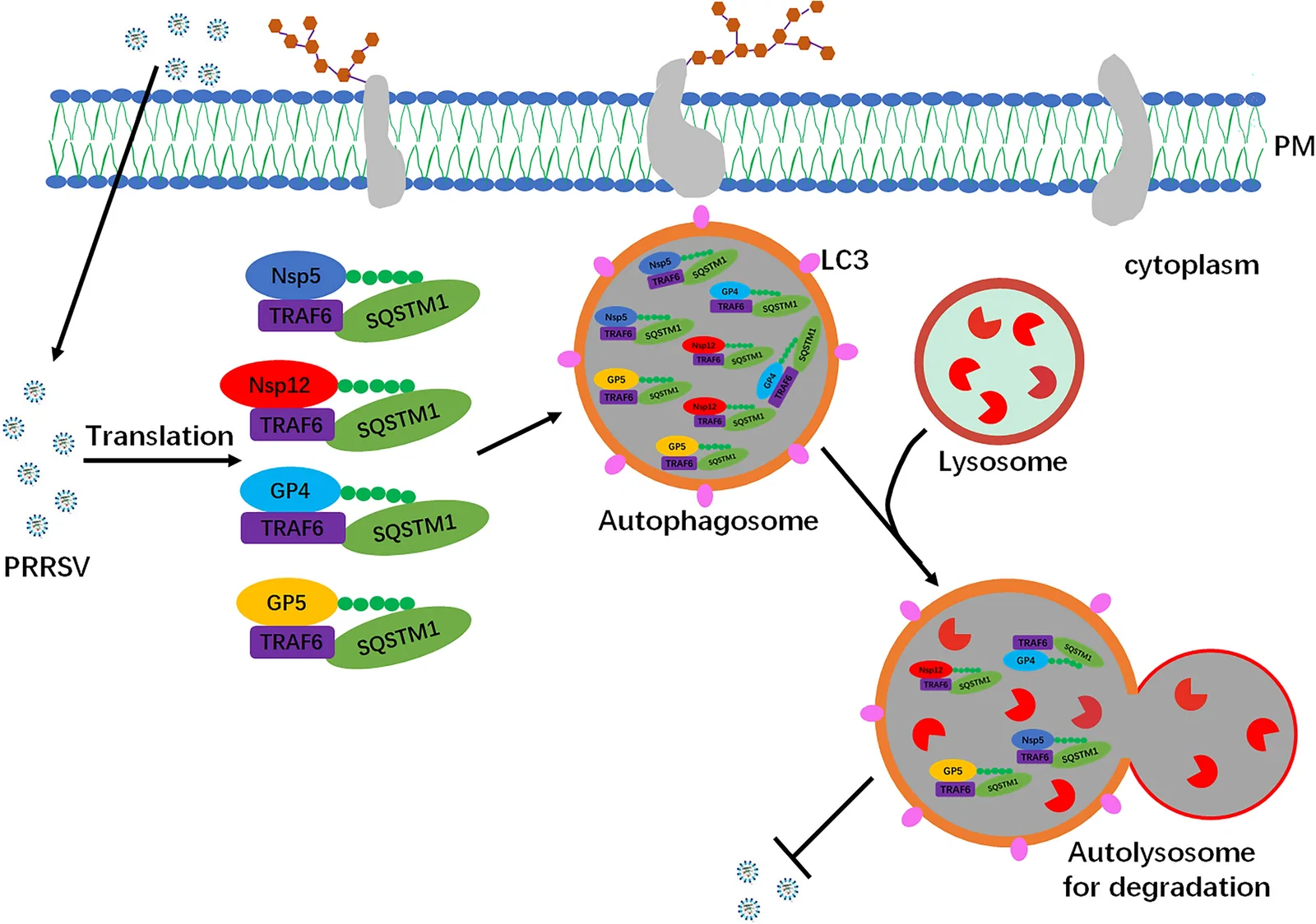
**Mechanisms underlying SQSTM1-mediated suppression of PRRSV replication.** SQSTM1 recruits TRAF6 to catalyze polyubiquitination of PRRSV Nsp5, Nsp12, GP4 and GP5, and subsequently targets these ubiquitinated viral proteins to autolysosomes for degradation, thereby suppressing PRRSV replication.

Similarly, we investigated the effect of TRAF6 on PRRSV replication. The results showed that overexpression of TRAF6 inhibited PRRSV N protein expression in a dose-dependent manner (Figure 7K), and reduced viral titers in the supernatant by 0.96 log_10_, 1.46 log_10_, and 2.45 log_10_, respectively (Figure 7L). Next, Marc-145 cells were co-transfected with Flag-TRAF6 and siNC or siSQSTM1, followed by infection with PRRSV at an MOI of 0.1. Western blot results showed that overexpression of Flag-TRAF6 inhibited PRRSV N protein expression; in contrast, knockdown of SQSTM1 partially reversed the antiviral activity of TRAF6 (Figure 7M). Consistent with this, TCID_50_ assay results indicated that overexpression of TRAF6 reduced viral titers in the supernatant by 1.63 log_10_ relative to the empty vector control group, whereas knockdown of SQSTM1 in the context of TRAF6 overexpression increased viral titers by 1.75 log_10_ relative to the siNC group (Figure 7N). Taken together, these findings demonstrate that SQSTM1 cooperates with TRAF6 to degrade PRRSV Nsp5, Nsp12, GP4, and GP5 proteins, thereby inhibiting viral replication (Figure 8).

## Discussion

Arteriviruses depend on the host machinery for their replication; meanwhile, they must interfere with the host’s defense system to achieve successful self-replication. This implies the existence of a highly complex virus-host interaction system, which may play a crucial role in the host’s defense against viral replication. The role of autophagy during PRRSV replication has been extensively studied; PRRSV can activate autophagy and utilize the autophagic pathway in diverse ways to facilitate its replication [37, 38, 51]. Moreover, pharmacological activation of the autophagic pathway promotes PRRSV replication [52]. In contrast, our results showed that induction of autophagic flux using the autophagy activator Rap suppresses PRRSV replication (data not shown). Considering that Rap induces the degradation of PRRSV key envelope protein GP4 (Figure 4E), this result is reasonable, indicating that the effect of autophagy on PRRSV replication is not conserved. Specifically, this may depend on the level of intracellular autophagic flux.

Appropriate autophagy is a prerequisite for host defense and immune responses [53]. Specifically, through the engagement of autophagy receptors, selective autophagy can function as an efficient defense mechanism against various viruses by degrading viral components or particles. For instance, SQSTM1 can degrade key proteins of dengue virus (DENV), Seneca Valley virus (SVV), porcine circovirus 2 (PCV2), and infectious bursal disease virus (IBDV) via the autophagic pathway, thereby suppressing viral replication. Meanwhile, aside from PRRSV, several other viruses have been shown to induce autophagy to benefit their replication [54]. It has been reported that flaviviruses exploit the lipid droplet protein AUP1 to trigger lipophagy and drive viral production [55]. These studies indicate the dual role of autophagy in host antiviral responses. In the present study, we demonstrated that SQSTM1 is a newly identified host restriction factor against PRRSV replication, which is associated with the autophagic degradation of viral Nsp5, Nsp12, GP4, and GP5 proteins. Notably, the mechanism by which SQSTM1 suppresses PRRSV replication is similar to its antiviral action against the aforementioned DENV, SVV, and IBDV. These findings suggest that selective autophagy plays a pivotal role in host defense against PRRSV replication.

In fact, the role of selective autophagy in host cell defense against PRRSV infection remains largely unknown. In contrast to non-selective autophagy, the process of selective autophagy mainly involves the recognition and degradation of ubiquitinated substrates by autophagy receptors, such as SQSTM1, NDP52, or NBR1 [56]. The present study showed that autophagy receptor SQSTM1 degrades the key envelope proteins GP4 and GP5 of PRRSV in a ubiquitin-dependent manner. The results confirm that host selective autophagy recognizes and degrades PRRSV components via substrate polyubiquitination. SQSTM1 binds to ubiquitinated proteins and delivers them to the autophagosome for final degradation in lysosomal compartments [57]. Further analysis showed that, by recruiting E3 ubiquitin ligase TRAF6, SQSTM1 facilitates the K63-linked polyubiquitination of GP4 and GP5, but not the K48-linked type. This is consistent with previous studies demonstrating that the K48-linked polyubiquitination typically targets proteins for proteasomal degradation, whereas K63-linked polyubiquitination primarily mediates degradation via the autophagic pathway [58–60]. Overexpression of SQSTM1 promotes the localization of PRRSV GP4 to lysosomal compartments, and the polyubiquitination of GP4 is required for SQSTM1-mediated degradation. This confirms that SQSTM1 acts as a bridge to facilitate the autophagy-lysosomal degradation of PRRSV GP4.

The present study also found that both PRRSV GP4 and GP5 proteins undergo polyubiquitination; however, the biological significance of the polyubiquitination of these key envelope proteins of PRRSV remains unclear. Previous studies have shown that the deubiquitination of PRRSV M and GP5 proteins, catalyzed by PRRSV deubiquitinase Nsp2, favors viral replication [61]. From this perspective, we speculate that the polyubiquitination of PRRSV GP4 and GP5, catalyzed by the host E3 ubiquitin ligase TRAF6, facilitates the degradation of viral proteins, representing a host strategy to defend against PRRSV infection. Overexpression of exogenous HA-tagged GP4 or HA-tagged GP5 alone resulted in the diffuse cytoplasmic distribution of both proteins, with no formation of large aggregate particles (Figure 4I). In contrast, solitary overexpression of Myc-tagged SQSTM1 led to its droplet-like localization in the cytoplasm (Figure 4I). When Myc-SQSTM1 was co-expressed with HA-GP4 or HA-GP5, SQSTM1 showed significant colocalization with GP4 or GP5, and both proteins exhibited a droplet-like distribution pattern (Figure 4I). Previous studies have established that polyubiquitin-induced SQSTM1 phase separation drives the concentration and segregation of autophagic cargo [62]. Collectively, these findings suggest that SQSTM1 may mediate the phase separation of ubiquitinated PRRSV envelope proteins GP4 and GP5 to facilitate their subsequent autophagic degradation, although this hypothesis requires further experimental validation.

SQSTM1 acts as a selective autophagy cargo receptor with multiple functional domains: the N-terminal PB1 domain mediates homo-oligomerization, the TB domain (a known TRAF6-binding region) recruits the E3 ubiquitin ligase TRAF6, the central LIR motif binds LC3, and the C-terminal UBA domain recognizes ubiquitinated substrates [30]. SQSTM1 coordinates the functions of these domains to mediate the autophagy–lysosomal degradation of ubiquitinated substrates and participates in host antiviral immunity. In this study, the UBA domain of SQSTM1 fails to interact with GP4; instead, SQSTM1 binds GP4 through its LIR motif. SQSTM1 further cooperates with LC3 to target GP4 to autophagosomes for degradation via the autophagy–lysosomal pathway, thereby suppressing PRRSV replication. This unconventional protein–protein interaction pattern may result from structural differences among distinct substrate proteins, which is structurally reasonable.

Covalent conjugation of ubiquitin or polyubiquitin chains to substrate proteins increases their apparent molecular mass during SDS-PAGE electrophoresis. In most proteins, K residues serve as the major ubiquitination sites. For PRRSV GP4 and GP5, sequential mutation of individual K residues to R abolished ubiquitination at these putative sites, which led to reduced apparent molecular masses of the mutant proteins relative to wild-type counterparts (Figure 6). Importantly, this electrophoretic mobility shift arises from changes in post-translational modification rather than differences in the theoretical molecular mass of the polypeptide backbone. Ubiquitination of viral proteins exerts multifaceted effects on viral pathogenicity. Viruses often exploit the host ubiquitin system to modify their own proteins to support viral assembly, trafficking and replication, thereby enhancing virulence. Meanwhile, ubiquitination also serves as a host defense signal: ubiquitin chains conjugated to viral proteins can be recognized by cargo receptors such as SQSTM1 to initiate selective autophagic degradation, restraining viral propagation [49]. The type of ubiquitin linkage dictates the biological outcome. In our study, TRAF6-mediated K63-linked polyubiquitination of PRRSV GP4 and GP5 acts as a molecular tag for SQSTM1 recognition and subsequent autophagic clearance. Mutation of these K sites abolishes ubiquitin modification and protects viral glycoproteins from SQSTM1-dependent degradation. Such alterations in viral protein stability may reshape PRRSV replication capacity and potentially modify its pathogenic phenotype in vivo. Although our in vitro data demonstrate that loss of these ubiquitination sites prevents GP4/GP5 from being targeted for autophagic degradation, how these mutations alter PRRSV virulence and pathogenicity in pigs remains to be characterized in animal challenge studies.

## Data Availability

No datasets were generated or analysed during the current study.

## References

[1] Zhang J, Bai J, Sun YY, Liu X, Gao YN, Wang XW, Yang YQ, Jiang P (2022) Comparison of pathogenicity of different subgenotype porcine reproductive and respiratory syndrome viruses isolated in China. Microb Pathog 168:105607 10.1016/j.micpath.2022.105607 35640766

[2] Wensvoort G, Terpstra C, Pol JMA, ter Laak EA, Bloemraad M, de Kluyver EP, Kragten C, van Buiten L, den Besten A, Wagenaar F, Broekhuijsen JM, Moonen PLJM, Zetstra T, de Boer EA, Tibben HJ, de Jong MF, van ‘t Veld P, Greenland GJR, van Gennep JA, Voets MT, Verheijden JHM, Braamskamp J (1991) Mystery swine disease in the Netherlands: the isolation of Lelystad virus. Vet Q 13:121–130 10.1080/01652176.1991.9694296 1835211

[3] Meulenberg JJ, Hulst MM, de Meijer EJ, Moonen PL, den Besten A, de Kluyver EP, Wensvoort G, Moormann RJ (1993) Lelystad virus, the causative agent of porcine epidemic abortion and respiratory syndrome (PEARS), is related to LDV and EAV. Virology 192:62–72 10.1006/viro.1993.1008 PMC7173055 8517032

[4] Lunney JK, Benfield DA, Rowland RRR (2010) Porcine reproductive and respiratory syndrome virus: an update on an emerging and re-emerging viral disease of swine. Virus Res 154:1–6 10.1016/j.virusres.2010.10.009 PMC7172856 20951175

[5] Li YH, Tas A, Sun Z, Snijder EJ, Fang Y (2015) Proteolytic processing of the porcine reproductive and respiratory syndrome virus replicase. Virus Res 202:48–59 10.1016/j.virusres.2014.12.027 25557977

[6] Snijder EJ, Dobbe JC, Spaan WJM (2003) Heterodimerization of the two major envelope proteins is essential for arterivirus infectivity. J Virol 77:97–104 10.1128/JVI.77.1.97-104.2003 PMC140607 12477814

[7] Wissink EHJ, Kroese MV, van Wijk HAR, Rijsewijk FAM, Meulenberg JJM, Rottier PJM (2005) Envelope protein requirements for the assembly of infectious virions of porcine reproductive and respiratory syndrome virus. J Virol 79:12495–12506 10.1128/JVI.79.19.12495-12506.2005 PMC1211556 16160177

[8] Lei XN, Jiang YF, Yu WT, Chen XY, Qin YW, Wang ND, Yang Y (2024) Intermolecular disulfide bond of PRRSV GP5 and M facilitates VLPs secretion and cell binding. Vet Microbiol 298:110249 10.1016/j.vetmic.2024.110249 39270333

[9] Wu WH, Fang Y, Rowland RRR, Lawson SR, Christopher-Hennings J, Yoon KJ, Nelson EA (2005) The 2b protein as a minor structural component of PRRSV. Virus Res 114:177–181 10.1016/j.virusres.2005.06.014 PMC7127422 16095746

[10] Durán MG, Costa S, Sarraseca J, de la Roja N, García J, García I, Rodríguez MJ (2016) Generation of porcine reproductive and respiratory syndrome (PRRS) virus-like-particles (VLPs) with different protein composition. J Virol Methods 236:77–86 10.1016/j.jviromet.2016.03.021 27435337

[11] Bai YZ, Sun Y, Liu YG, Zhang HL, An TQ, Wang Q, Tian ZJ, Qiao XY, Cai XH, Tang YD (2024) Minor envelope proteins from GP2a to GP4 contribute to the spread pattern and yield of type 2 PRRSV in MARC-145 cells. Front Cell Infect Mi 14:1376725 10.3389/fcimb.2024.1376725 PMC10999527 38590440

[12] Guo H, Gaowa W, Zhao HZ, Liu CY, Hou LA, Wen YJ, Wang FX (2023) Glycosylated protein 4-deficient PRRSV in complementing cell line shows low virus titer. Res Vet Sci 158:84–95 10.1016/j.rvsc.2023.03.010 36958176

[13] Costers S, Vanhee M, Van Breedam W, Van Doorsselaere J, Geldhof M, Nauwynck HJ (2010) GP4-specific neutralizing antibodies might be a driving force in PRRSV evolution. Virus Res 154:104–113 10.1016/j.virusres.2010.08.026 20837070

[14] Kimpston-Burkgren K, Correas I, Osorio FA, Steffen D, Pattnaik AK, Fang Y, Vu HLX (2017) Relative contribution of porcine reproductive and respiratory syndrome virus open reading frames 2–4 to the induction of protective immunity. Vaccine 35:4408–4413 10.1016/j.vaccine.2017.06.061 28689650

[15] An CH, Nazki S, Park SC, Jeong YJ, Lee JH, Park SJ, Khatun A, Kim WI, Park YI, Jeong JC, Kim CY (2018) Plant synthetic GP4 and GP5 proteins from porcine reproductive and respiratory syndrome virus elicit immune responses in pigs. Planta 247:973–985 10.1007/s00425-017-2836-z 29313103

[16] Chen X, Zhao JK, Ji PP, Li XX, Niu H, Jiao D, Zhang L, Zhu QY, Liu XY, Hiscox JA, Stewart JP, Sun YN, Zhao Q (2025) A porcine monoclonal antibody targeting a conserved GP4 epitope protects against in vivo infection via the induction of broad-spectrum PRRSV neutralization. Adv Sci 12:e08875 10.1002/advs.202508875 PMC12667497 40919718

[17] Du YJ, Pattnaik AK, Song C, Yoo DW, Li G (2012) Glycosyl-phosphatidylinositol (GPI)-anchored membrane association of the porcine reproductive and respiratory syndrome virus GP4 glycoprotein and its co-localization with CD163 in lipid rafts. Virology 424:18–32 10.1016/j.virol.2011.12.009 PMC7111931 22222209

[18] Wang L, Li R, Geng R, Zhang LX, Chen XX, Qiao SL, Zhang GP (2022) Heat shock protein member 8 (HSPA8) is involved in porcine reproductive and respiratory syndrome virus attachment and internalization. Microbiol Spectr 10:e0186021 10.1128/spectrum.01860-21 PMC8826899 35138165

[19] Delputte PL, Costers S, Nauwynck HJ (2005) Analysis of porcine reproductive and respiratory syndrome virus attachment and internalization: distinctive roles for heparan sulphate and sialoadhesin. J Gen Virol 86:1441–1445 10.1099/vir.0.80675-0 15831956

[20] Van Breedam W, Van Gorp H, Zhang JQ, Crocker PR, Delputte PL, Nauwynck HJ (2010) The M/GP glycoprotein complex of porcine reproductive and respiratory syndrome virus binds the sialoadhesin receptor in a sialic acid-dependent manner. PLoS Pathog 6:e1000730 10.1371/journal.ppat.1000730 PMC2799551 20084110

[21] Zhang MZ, Han XL, Osterrieder K, Veit M (2021) Palmitoylation of the envelope membrane proteins GP5 and M of porcine reproductive and respiratory syndrome virus is essential for virus growth. PLoS Pathog 17:e1009554 10.1371/journal.ppat.1009554 PMC8099100 33891658

[22] Das PB, Dinh PX, Ansari IH, de Lima M, Osorio FA, Pattnaik AK (2010) The minor envelope glycoproteins GP2a and GP4 of porcine reproductive and respiratory syndrome virus interact with the receptor CD163. J Virol 84:1731–1740 10.1128/JVI.01774-09 PMC2812361 19939927

[23] Münz C, Campbell GR, Esclatine A, Faure M, Labonte P, Lussignol M, Orvedahl A, Altan-Bonnet N, Bartenschlager R, Beale R, Cirone M, Espert L, Jung J, Leib D, Reggiori F, Sanyal S, Spector SA, Thiel V, Viret C, Wei Y, Wileman T, Wodrich H (2025) Autophagy machinery as exploited by viruses. Autophagy Rep 4:2464986 10.1080/27694127.2025.2464986 PMC11921968 40201908

[24] Johansen T, Lamark T (2011) Selective autophagy mediated by autophagic adapter proteins. Autophagy 7:279–296 10.4161/auto.7.3.14487 PMC3060413 21189453

[25] Rogov V, Dötsch V, Johansen T, Kirkin V (2014) Interactions between autophagy receptors and ubiquitin-like proteins form the molecular basis for selective autophagy. Mol Cell 53:167–178 10.1016/j.molcel.2013.12.014 24462201

[26] Pankiv S, Clausen TH, Lamark T, Brech A, Bruun JA, Outzen H, Overvatn A, Bjorkoy G, Johansen T (2007) p62/SQSTM1 binds directly to Atg8/LC3 to facilitate degradation of ubiquitinated protein aggregates by autophagy. J Biol Chem 282:24131–24145 10.1074/jbc.M702824200 17580304

[27] Kirkin V, Lamark T, Sou YS, Bjorkoy G, Nunn JL, Bruun JA, Shvets E, McEwan DG, Clausen TH, Wild P, Bilusic I, Theurillat JP, Overvatn A, Ishii T, Elazar Z, Komatsu M, Dikic I, Johansen T (2009) A role for NBR1 in autophagosomal degradation of ubiquitinated substrates. Mol Cell 33:505–516 10.1016/j.molcel.2009.01.020 19250911

[28] von Muhlinen N, Thurston T, Ryzhakov G, Bloor S, Randow F (2010) NDP52, a novel autophagy receptor for ubiquitin-decorated cytosolic bacteria. Autophagy 6:288–289 10.4161/auto.6.2.11118 20104023

[29] Kirkin V, Rogov VV (2019) A diversity of selective autophagy receptors determines the specificity of the autophagy pathway. Mol Cell 76:268–285 10.1016/j.molcel.2019.09.005 31585693

[30] Moscat J, Diaz-Meco MT (2009) p62 at the crossroads of autophagy, apoptosis, and cancer. Cell 137:1001–1004 10.1016/j.cell.2009.05.023 PMC3971861 19524504

[31] Adriaenssens E, Ferrari L, Martens S (2022) Orchestration of selective autophagy by cargo receptors. Curr Biol 32:R1357–R1371 10.1016/j.cub.2022.11.002 36538890

[32] Li Z, Lai YN, Qiu RH, Tang WB, Ren J, Xiao SB, Fang PX, Fang LR (2024) Hyperacetylated microtubules assist porcine deltacoronavirus nsp8 to degrade MDA5 via SQSTM1/p62-dependent selective autophagy. J Virol 98:e0000324 10.1128/jvi.00003-24 PMC10949429 38353538

[33] Wang XQ, Tong W, Yang XY, Zhai HJ, Qin WZ, Liu CL, Zheng H, Yu H, Tong GZ, Zhang ZD, Kong N, Shan TL (2024) RBM14 inhibits the replication of porcine epidemic diarrhea virus by recruiting p62 to degrade nucleocapsid protein through the activation of autophagy and interferon pathway. J Virol 98:e0018224 10.1128/jvi.00182-24 PMC10949495 38411947

[34] Xu CY, Li TT, Lei J, Zhang YA, Zhou JY, Hu BL (2021) The autophagy cargo receptor SQSTM1 inhibits infectious bursal disease virus infection through selective autophagic degradation of double-stranded viral RNA. Viruses 13:2494 10.3390/v13122494 PMC8704251 34960763

[35] Guo JS, Shi YY, Jiang GH, Zeng PH, Wu Z, Wang DD, Cui YQ, Yang XY, Zhou JW, Feng XF, Hou L, Liu J (2024) SQSTM1 downregulates avian metapneumovirus subgroup C replication via mediating selective autophagic degradation of viral M2–2 protein. J Virol 98:e0005124 10.1128/jvi.00051-24 PMC11019959 38466095

[36] Li YH, Hu BL, Ji G, Zhang YN, Xu CY, Lei J, Ding C, Zhou JY (2020) Cytoplasmic cargo receptor p62 inhibits avibirnavirus replication by mediating autophagic degradation of viral protein VP2. J Virol 94:10 10.1128/JVI.01255-20 PMC7925189 32967959

[37] Diao FF, Jiang CL, Sun YY, Gao YN, Bai J, Nauwynck H, Wang XW, Yang YQ, Jiang P, Liu X (2023) Porcine reproductive and respiratory syndrome virus infection triggers autophagy via ER stress-induced calcium signaling to facilitate virus replication. PLoS Pathog 19:e1011295 10.1371/journal.ppat.1011295 PMC10079224 36972295

[38] Sun MX, Huang L, Wang R, Yu YL, Li C, Li PP, Hu XC, Hao HP, Ishag HA, Mao X (2012) Porcine reproductive and respiratory syndrome virus induces autophagy to promote virus replication. Autophagy 8:1434–1447 10.4161/auto.21159 22739997

[39] Liu QH, Qin YX, Zhou L, Kou QW, Guo X, Ge XN, Yang HC, Hu HB (2012) Autophagy sustains the replication of porcine reproductive and respiratory virus in host cells. Virology 429:136–147 10.1016/j.virol.2012.03.022 PMC7111961 22564420

[40] Zhang W, Chen KR, Guo Y, Chen YS, Liu XH (2019) Involvement of PRRSV NSP3 and NSP5 in the autophagy process. Virol J 16:13 10.1186/s12985-019-1116-x PMC6350329 30691473

[41] Lu LC, Wang QM, Yi HY, Tian T, Chen AL, Zheng GX, Ye RR, Xie ER, Zhang GH, Wang H (2025) Porcine reproductive and respiratory syndrome virus Nsp2 induces incomplete autophagy to facilitate viral replication via disrupting SNARE complex assembly. Int J Biol Macromol 331:148288 10.1016/j.ijbiomac.2025.148288 41093191

[42] Li W, Zhang MT, Wang YS, Zhao SJ, Xu PL, Cui ZY, Chen J, Xia PA, Zhang YN (2024) PRRSV GP5 inhibits the antivirus effects of chaperone-mediated autophagy by targeting LAMP2A. MBio 15:e0053224 10.1128/mbio.00532-24 PMC11323736 38940560

[43] Dong HXPQ, Ren JH, Zhang YC, Wei XD, Shen AJ, Lu YS, Zhang ZH, Du YK, Zhuang GQ, Zhang AK, Duan H (2025) Cholesterol-dependent Nsp5-endosomes co-trafficking to lysosomes facilitates porcine reproductive and respiratory syndrome virus replication by activating autophagy. Vet Microbiol 305:110507 10.1016/j.vetmic.2025.110507 40215803

[44] Chen BH, Luo YY, Chen YJ, Wang JX, Yan JC, He Z, Li FF, Guo CH (2025) The DEAD-box RNA helicase 27 negatively regulates the replication of porcine reproductive and respiratory syndrome virus by mediating GP2a autophagy degradation and inducing interferon-β production. Front Immunol 16:1587647 10.3389/fimmu.2025.1587647 PMC12197943 40574840

[45] Li LW, Bai YZ, Zhou YJ, Jiang YF, Tong W, Li GX, Zheng HH, Gao F, Tong GZ (2023) PSMB1 inhibits the replication of porcine reproductive and respiratory syndrome virus by recruiting NBR1 to degrade nonstructural protein 12 by autophagy. J Virol 97:e0166022 10.1128/jvi.01660-22 PMC9888268 36602366

[46] Yi HY, Wang QM, Lu LC, Ye RR, Xie E, Yu ZQ, Sun YK, Chen Y, Cai MK, Qiu YW, Wu QW, Peng J, Wang H, Zhang GH (2023) PSMB4 degrades the porcine reproductive and respiratory syndrome virus Nsp1α protein via the autolysosome pathway and induces the production of type I interferon. J Virol 97:e0026423 10.1128/jvi.00264-23 PMC10134815 36943051

[47] Duan HDH, Wu SY, Ren JH, Zhang MF, Chen CW, Du YK, Zhang GP, Zhang AK (2022) Porcine reproductive and respiratory syndrome virus non-structural protein 4 cleaves guanylate-binding protein 1 via its cysteine proteinase activity to antagonize GBP1 antiviral effect. Vet Res 53:55 10.1186/s13567-022-01071-8 PMC9264745 35804432

[48] Turco ESA, Gere F, Ferrari L, Romanov J, Schuschnig M, Martens S (2021) Reconstitution defines the roles of p62, NBR1 and TAX1BP1 in ubiquitin condensate formation and autophagy initiation. Nat Commun 12:5212 10.1038/s41467-021-25572-w PMC8410870 34471133

[49] Viret C, Duclaux-Loras R, Nancey S, Rozières A, Faure M (2021) Selective autophagy receptors in antiviral defense. Trends Microbiol 29:798–810 10.1016/j.tim.2021.02.006 33678557

[50] Berkamp S, Mostafavi S, Sachse C (2021) Structure and function of p62/SQSTM1 in the emerging framework of phase separation. FEBS J 288:6927–6941 10.1111/febs.15672 33332721

[51] Zhao SS, Qian QS, Chen XX, Lu QX, Xing GX, Qiao SL, Li R, Zhang GP (2024) Porcine reproductive and respiratory syndrome virus triggers Golgi apparatus fragmentation-mediated autophagy to facilitate viral self-replication. J Virol 98:e01842 10.1128/jvi.01842-23 PMC10878038 38179942

[52] Chen QG, Fang LR, Wang D, Wang SH, Li P, Li M, Luo R, Chen HC, Xiao SB (2012) Induction of autophagy enhances porcine reproductive and respiratory syndrome virus replication. Virus Res 163:650–655 10.1016/j.virusres.2011.11.008 PMC7114478 22119900

[53] Deretic V, Saitoh T, Akira S (2013) Autophagy in infection, inflammation and immunity. Nat Rev Immunol 13:722–737 10.1038/nri3532 PMC5340150 24064518

[54] Lv JM, Jiang YF, Feng QW, Fan ZX, Sun Y, Xu PP, Hou YF, Zhang XP, Fan YX, Xu XG, Zhang YM, Guo KK (2020) Porcine circovirus type 2 ORF5 protein induces autophagy to promote viral replication via the PERK-eIF2α-ATF4 and mTOR-ERK1/2-AMPK signaling pathways in PK-15 cells. Front Microbiol 11:320 10.3389/fmicb.2020.00320 PMC7058596 32184774

[55] Zhang JS, Lan Y, Li MY, Lamers MM, Fusade-Boyer M, Klemm E, Thiele C, Ashour J, Sanyal S (2018) Flaviviruses exploit the lipid droplet protein AUP1 to trigger lipophagy and drive virus production. Cell Host Microbe 23:819–831 10.1016/j.chom.2018.05.005 29902443

[56] Mello-Vieira JD (2026) Ubiquitination and autophagy in host-pathogen interactions: from immune surveillance to therapeutic targeting. Nat Rev Immunol 26:335–349 10.1038/s41577-025-01239-1 41491841

[57] Vadlamudi RK, Joung I, Strominger JL, Shin J (1996) p62, a phosphotyrosine-independent ligand of the SH2 domain of p56, belongs to a new class of ubiquitin-binding proteins. J Biol Chem 271:20235–20237 10.1074/jbc.271.34.20235 8702753

[58] Kirkin V, McEwan DG, Novak I, Dikic I (2009) A role for ubiquitin in selective autophagy. Mol Cell 34:259–269 10.1016/j.molcel.2009.04.026 19450525

[59] Tan JMM, Wong ESP, Kirkpatrick DS, Pletnikova O, Ko HS, Tay SP, Ho MWL, Troncoso J, Gygi SP, Lee MK, Dawson VL, Dawson TM, Lim KL (2008) Lysine 63-linked ubiquitination promotes the formation and autophagic clearance of protein inclusions associated with neurodegenerative diseases. Hum Mol Genet 17:431–439 10.1093/hmg/ddm320 17981811

[60] Rui YN, Xu Z, Patel B, Chen ZH, Chen DS, Tito A, David G, Sun YM, Stimming EF, Bellen HJ, Cuervo AM, Zhang S (2015) Huntingtin functions as a scaffold for selective macroautophagy. Nat Cell Biol 17:262–275 10.1038/ncb3101 PMC4344873 25686248

[61] Guo R, Yan XY, Li YH, Cui J, Misra S, Firth AE, Snijder EJ, Fang Y (2021) A swine arterivirus deubiquitinase stabilizes two major envelope proteins and promotes production of viral progeny. PLoS Pathog 17:e1009403 10.1371/journal.ppat.1009403 PMC7971519 33735221

[62] Sun DX, Wu RB, Zheng JX, Li PL, Yu L (2018) Polyubiquitin chain-induced p62 phase separation drives autophagic cargo segregation. Cell Res 28:405–415 10.1038/s41422-018-0017-7 PMC5939046 29507397

